# Novel Chromosome Organization Pattern in *Actinomycetales*—Overlapping Replication Cycles Combined with Diploidy

**DOI:** 10.1128/mBio.00511-17

**Published:** 2017-06-06

**Authors:** Kati Böhm, Fabian Meyer, Agata Rhomberg, Jörn Kalinowski, Catriona Donovan, Marc Bramkamp

**Affiliations:** a Ludwig-Maximilians-Universität München, Fakultät Biologie, Planegg-Martinsried, Germany; b Universität Bielefeld, Center for Biotechnology (CeBiTec), Bielefeld, Germany; Nanyang Technological University

**Keywords:** *Corynebacterium*, ParA, ParB, cell cycle, diploidy, origin, replication

## Abstract

Bacteria regulate chromosome replication and segregation tightly with cell division to ensure faithful segregation of DNA to daughter generations. The underlying mechanisms have been addressed in several model species. It became apparent that bacteria have evolved quite different strategies to regulate DNA segregation and chromosomal organization. We have investigated here how the actinobacterium *Corynebacterium glutamicum* organizes chromosome segregation and DNA replication. Unexpectedly, we found that *C. glutamicum* cells are at least diploid under all of the conditions tested and that these organisms have overlapping C periods during replication, with both origins initiating replication simultaneously. On the basis of experimental data, we propose growth rate-dependent cell cycle models for *C. glutamicum*.

## IMPORTANCE

Bacterial cell cycles are known for few model organisms and can vary significantly between species. Here, we studied the cell cycle of *Corynebacterium glutamicum*, an emerging cell biological model organism for mycolic acid-containing bacteria, including mycobacteria. Our data suggest that *C. glutamicum* carries two pole-attached chromosomes that replicate with overlapping C periods, thus initiating a new round of DNA replication before the previous one is terminated. The newly replicated origins segregate to midcell positions, where cell division occurs between the two new origins. Even after long starvation or under extremely slow-growth conditions, *C. glutamicum* cells are at least diploid, likely as an adaptation to environmental stress that may cause DNA damage. The cell cycle of *C. glutamicum* combines features of slow-growing organisms, such as polar origin localization, and fast-growing organisms, such as overlapping C periods.

## INTRODUCTION

Bacterial chromosome organization is highly regulated, where replication coincides with the segregation of sister nucleoids and is tightly coordinated with cell division (1). Cell cycle control mechanisms exist that ensure constant DNA content throughout cell generations. In particular, the action of the key replication initiator protein DnaA is timed by various regulatory systems, for instance, via the CtrA protein cascade in *Caulobacter crescentus* or SeqA in *Escherichia coli* (2–6). Upon replication initiation, DnaA binds to the origin of replication (oriC) and mediates duplex unwinding prior to loading of the replication machinery (7, 8). The two evolving replication forks migrate along the left and right arms of the circular chromosome toward the terminus of replication (terC), where (FtsK-dependent) XerCD recombinases resolve chromosome dimers as a final step, as shown in *E. coli* (9, 10). Replication usually takes place within defined cellular regions via stably assembled protein complexes, namely, replisomes, of a rather static or dynamic nature (11, 12).

**FIG 1:**
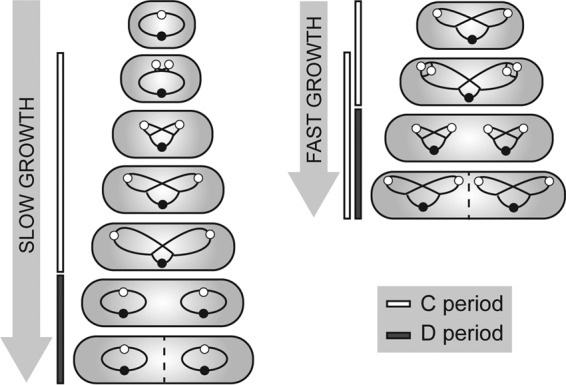
Schematic representation of bacterial replication cycles under slow (left)- and fast (right)-growth conditions. Under slow-growth conditions, DNA replication (termed the C period) takes place within a single generation, followed by the interval between replication termination and completion of cell division (the D period). Fast-growing bacteria with generation times shorter than the C period, like *B. subtilis* and *E. coli*, undergo multifork replication; i.e., new rounds of replication are initiated before previous ones terminate. Chromosomes are indicated by black lines with oriCs and terCs as white and black circles.

The bacterial cell cycle can be divided into different stages, as illustrated in Fig. 1. The time of DNA replication is termed the C period. It is followed by a time interval necessary for cell division executed by the divisome (the D period). Several bacteria, like *Mycobacterium smegmatis* and *C. crescentus*, replicate their genomes once within a generation, where the C periods are temporally separated from each other (13, 14). Under slow-growth conditions, a nonreplicative state termed the B period precedes the C period (not shown); thus, the bacterial cell cycle resembles the eukaryotic cell cycle in some aspects (G_1_, S, and G_2_ phases). Contrary to this, fast-growing organisms such as *Bacillus subtilis*, *E. coli*, and *Vibrio cholerae* can overlap C periods during fast growth, a phenomenon termed multifork replication (15–17). Under these conditions, a new round of replication is reinitiated before the termination of the previous one. Therefore, generation times are considerably shorter than the duration of the C period. However, only one round of replication is initiated per cell cycle and usually one C period is completed at the time point of cell division (18). Many bacteria contain only one copy of the chromosome. However, several bacteria and archaea can have increased DNA contents because of oligo- or polyploidy (19). Polyploid cells harbor multiple, fully replicated chromosome copies throughout their life cycle, which has been frequently found in prokaryotes, including certain Gram-positive bacteria, proteobacteria, members of the order *Deinococcales*, cyanobacteria, and also archaea (20–27).

Besides the distinct cell cycle modes, chromosome localization patterns differ between model organisms. In a nonreplicating, slow-growing *E. coli* cell, the single chromosome is placed symmetrically, with the oriC and terC regions located at midcell positions and the replichores spatially separated in the two cell halves (28). Upon replication initiation, the two sister chromosomes segregate bidirectionally to opposite cell halves, with replisomes positioned at the midcell position (29, 30). Finally, oriC and terC are confined to cell quarter regions. Contrary to this, the model organisms *C. crescentus*, *V. cholerae*, and *Pseudomonas aeruginosa* localize their nucleoids about the longitudinal axis with chromosome arms adjacent to each other (31–34). Sister replichores move to the opposite cell half, with the segregated oriC facing toward the pole, mirroring the second chromosome at the transverse axis. The oriC region of *C. crescentus* and *V. cholerae* is positioned by polar landmark proteins (35, 36), where replisomes assemble and simultaneously move toward the midcell position in the course of replication (12, 17). For the most part, *P. aeruginosa* places its replication machinery centrally (34). Finally, *B. subtilis* switches from longitudinal chromosome organization to the *E. coli* left-oriC-right configuration during replication initiation (37).

The mitotic-like ParABS segregation system has been identified as a driving force behind coordinated nucleoid partitioning for more than two-thirds of the bacterial species analyzed, with exceptions specifically within the class *Gammaproteobacteria*, such as *E. coli* (38). This segregation mechanism involves components similar to the plasmid-located *par* genes responsible for active segregation of low-copy-number plasmids (39). Thereby, the ParB protein binds a variable number of centromere-like DNA sequences called *parS* sites in the proximity of oriC (40) and spreads along the DNA, forming large protein-DNA complexes (41–43). Interaction of ParB with the Walker-type ATPase ParA mediates ATP hydrolysis and thereby ParA detachment from DNA (44), driving apart the sister chromosomes as the ParA-ParB interaction translocates oriC toward the opposite cell half (33, 45, 46). The precise mechanism of ParABS-mediated DNA segregation has been under debate; however, to date, dynamic diffusion ratchet and DNA relay models are favored, where nucleoid and plasmid movement is mediated along a ParA gradient caused by local ParB-stimulated depletion of DNA-bound ParA (47–49). Deletion of this partitioning system has mild effects in *B. subtilis* and *V. cholerae* cells but causes severe chromosome segregation defects in other organisms and is essential for viability in *C. crescentus* and *Myxococcus xanthus* (46, 50–56).

Here we present the cell cycle and spatiotemporal organization of oriCs and replisomes in *C. glutamicum*, a rod-shaped, polar-growing actinobacterium. It is closely related to pathogens like *Corynebacterium diphtheriae* and *Mycobacterium tuberculosis*, the latter being among the top 10 causes of fatal infections worldwide (57). Besides this, *C. glutamicum* is of great economic importance as an amino acid and vitamin producer and extensive efforts in metabolic engineering are being carried out concerning metabolite production and yield increase (58). Although its metabolism is one of the best studied among model organisms, the underlying cell cycle parameters and chromosome organization patterns have not previously been analyzed in detail. *C. glutamicum* relies on a ParABS system to segregate its nucleoids prior to cell division (51, 59, 60). Chromosome segregation influences division site selection, and hence, growth and chromosome organization are tightly coupled in *C. glutamicum* (59). This may, in part, explain why protein machineries that have been described in various bacterial species like the Min system or a nucleoid occlusion system, both of which are involved in division septum placement, are absent from *C. glutamicum* (61).

In this study, we tracked *in vivo* fluorescently labeled centromere-binding protein ParB, the terminus region of the chromosome, and the replisome sliding-clamp DnaN to investigate spatiotemporal oriC, terC, and replisome localization throughout the cell cycle. Fluorescence microscopy and single-cell tracking by time-lapse analysis revealed remarkably high oriC and replisome numbers during fast growth, suggesting multiple chromosomes and several simultaneous replication events per cell. Initially, cells possess two polar oriC-ParB clusters whereby, upon replication, sister oriCs segregate toward midcell positions. Additionally, the length of replication periods, as well as the overall DNA content, was determined for different growth conditions by marker frequency analysis and flow cytometry, thereby allowing the formulation of complete cell cycle models. Our data suggest diploidy and overlapping C periods in *C. glutamicum* and therefore give new insights into replication coordination within the class *Actinobacteria*.

## RESULTS

### Origin numbers correlate with cell length in a ParA-independent way.

**FIG 2:**
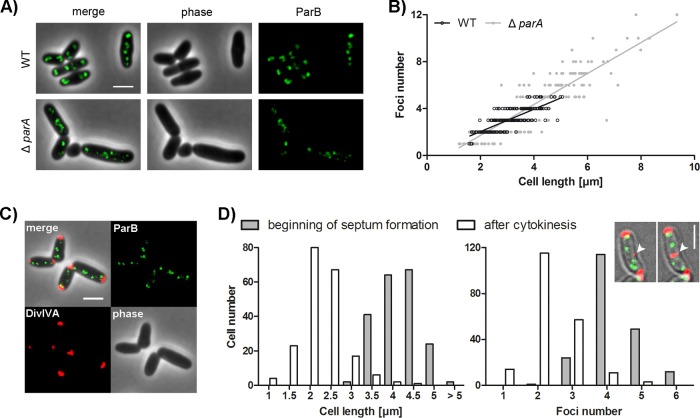
Determination of the *C. glutamicum* oriC number and correlation with cell length. (A) Subcellular localization of ParB-eYFP in representative *parB*::*parB-eYFP* WT and Δ*parA* mutant cells. Shown are overlays of phase-contrast images and eYFP fluorescence (merge) and separate channels (phase, ParB). Scale bar, 2 µm. (B) ParB focus number depends on cell length in a ParA-independent way. In the WT strain, 1 to 5 foci were observed, and in a Δ*parA* mutant strain, 1 to 12 foci were observed (*n* = 400). Linear regression lines are shown. WT, *r* = 0.80; Δ*parA* mutant, *r* = 0.88; slopes are not equal (ANCOVA, *F*^1,396^ = 16.10, *P* < 0.0001). (C) Still image of *C. glutamicum parB*::*parB-eYFP divIVA*::*divIVA-mCherry* cells. Depicted are a phase-contrast image (lower right), DivIVA (lower left) and ParB-eYFP (upper right) fluorescence, and an overlay of all of the channels (upper left). Scale bar, 2 µm. (D) Time-lapse microscopy of a DivIVA-mCherry- and ParB-eYFP-coexpressing strain reveals distribution of cell length and ParB-eYFP cluster number at the beginning of septum formation and after cell division (*n* = 200). The microscopy images of a single cell exemplify the time-lapse analysis of septum formation (white arrowheads) tracked with a DivIVA-mCherry reporter. Scale bar, 2 µm.

We have shown before that the *C. glutamicum* partitioning protein ParB localizes at the origin regions of the chromosome close to the cell poles (51). However, in-depth studies of spatiotemporal chromosome organization were still missing. Therefore, we aimed to reanalyze oriC-ParB complexes microscopically by time-resolved live-cell imaging. To label origin regions, the native chromosomal *parB* gene of wild-type (WT) or Δ*parA* mutant *C. glutamicum* RES167 was replaced with *parB-eYFP*, resulting in strains with typical cell morphology and growth phenotypes (Fig. 2A; see Fig. S1 in the supplemental material). No cleavage products of ParB-eYFP were detectable (see Fig. S1), suggesting that fluorescent signals faithfully reflect ParB localization. Microscopic analysis revealed a correlation of ParB-eYFP focus numbers with cell length in both strains (Fig. 2B). In the WT background, between one and five foci were detected. The *parA* deletion mutant has variable cell lengths, and anucleate minicells (not taken into account) were observed. Up to 12 ParB foci were present in cells of the *parA* deletion mutant. Notably, most of the ParB foci in the *parA* knockout strain were less fluorescent than in WT cells, suggesting that lack of ParA causes problems in oriC-ParB assembly at the cell poles. Chromosome segregation defects upon *parA* deletion do not markedly affect the high correlation of the oriC-ParB focus number with cell length. However, linear regression models yield significant differences between the WT and *parA* deletion strains (Fig. 2B). Notably, the ParB concentration is not increased in the absence of *parA* (see Fig. S1D). The higher oriC numbers could be caused by a loss of oriC-ParB complex cohesion or reduced tethering to cell poles, which might be positively influenced by ParA.

10.1128/mBio.00511-17.2 FIG S1  Growth and cell length distributions of the strains used in this work. (A, B) Comparison of growth curves (A) and cell length distributions (B) (*n* = 200) of *C. glutamicum* mutant strains with those of the WT. All growth experiments were performed with BHI medium. Values were derived from triplicate measurements; standard deviations are indicated in all graphs. *C. glutamicum parB*::*parB-eYFP*, *parB*::*parB-eYFP divIVA*::*divIVA-mCherry*, *dnaN*::*dnaN-mCherry*, *parB*::*parB-mCherry2*, *parB*::*parB-eYFP dnaN*::*dnaN-mCherry*, Δ*parA*, and Δ*parA parB*::*parB-eYFP* mutant strains were analyzed. (C) Validation of full-length fusion proteins ParB-mCherry2 and DnaN-mCherry. Full-length fluorescent fusion proteins were validated via Western blotting. Whole-cell lysates of WT (lanes 1) and *dnaN*::*dnaN-mCherry* (lanes 2) and *parB*::*parB-mCherry2* (lanes 3) mutant *C. glutamicum* strains were probed, and DnaN-mCherry and ParB-mCherry2 were detected via anti-mCherry polyclonal antibody. Shown are a Western blot analysis (left) and the corresponding Coomassie-stained gel (right). (D) Similar ParB-mCherry2 protein levels in the WT and Δ*parA* mutant backgrounds. Amounts of full-length ParB-mCherry2 fusion proteins in WT (lanes 1) and *parB*::*parB-mCherry2* (lanes 2) and Δ*parA parB*::*parB-mCherry2* (lanes 3) mutant *C. glutamicum* cells were visualized via Western blotting (left). The total protein load is shown by Coomassie staining (right). Download FIG S1, TIF file, 0.9 MB. Copyright © 2017 Böhm et al. 2017 Böhm et al. This content is distributed under the terms of the Creative Commons Attribution 4.0 International license.

To determine the maximal number of oriCs per cell precisely, an additional allelic replacement of *divIVA* with *divIVA-mCherry* was carried out (Fig. 2C). With this construct, cell division can be monitored by DivIVA localization before the walls of the daughter cells are separated, because DivIVA efficiently accumulates at the septal membrane. This strain revealed WT-like growth rates and cell length distributions (see Fig. S1), suggesting that DivIVA-mCherry and ParB-eYFP are functional. DivIVA localizes to the cell poles, as well as newly formed septa, and therefore is a suitable marker for completed cell division (60). Analysis revealed an average of four ParB foci prior to completed septation at an average cell length of 3.94 µm; however, up to six foci per cell could be determined (Fig. 2D). Newborn cells with an average length of 2.01 µm mostly contained two foci. Origin numbers and cell size were relatively variable, as division septa are often not precisely placed at the midcell position in corynebacteria. Use of the DivIVA reporter to judge the ParB focus number per daughter cell resulted in oriC numbers per cell similar to those obtained when using the physical separation of daughter cells as a marker of cell division. Notably, these analyses revealed unexpected high oriC-ParB cluster numbers that hint at the ability of *C. glutamicum* to undergo multifork replication and/or to harbor multiple fully replicated chromosomes per cell.

### **Spatiotemporal localization pattern of ParB**-**origin complexes.**

**FIG 3:**
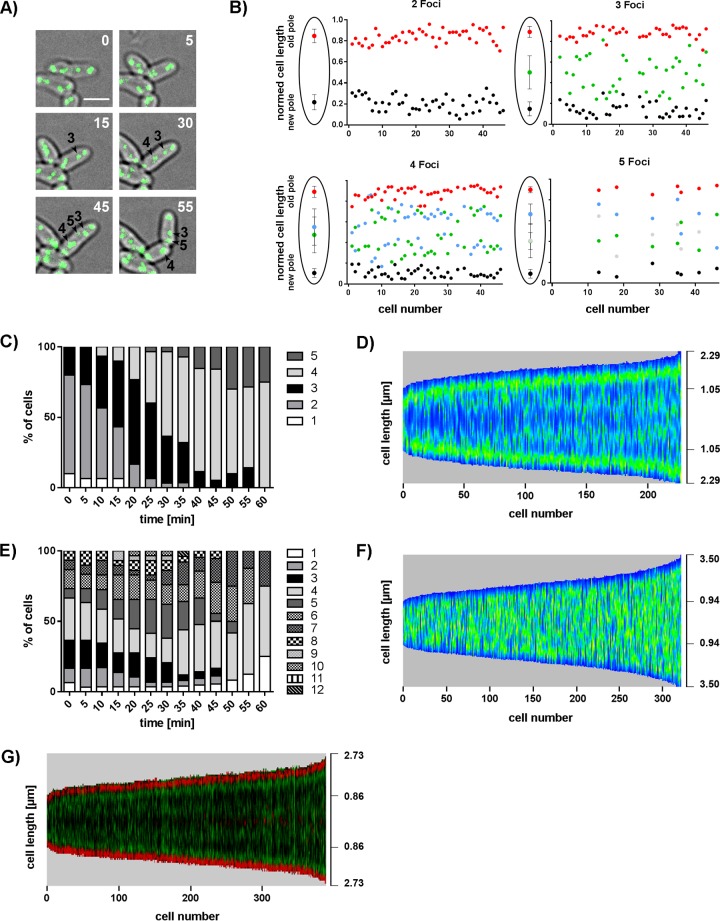
oriC localization pattern during cell cycle progression. (A) Occurrence of newly formed ParB-eYFP clusters in the course of cell elongation. Still images show a time series of a typical *C. glutamicum* WT cell with initially two ParB spots. Three further foci appear over time (arrowheads); time points are indicated in minutes (top right corners). Scale bar, 2 µm. (B) Time-lapse single-cell analyses reveal oriC-ParB complex positions along the long cell axis at each moment in time when a new ParB-eYFP spot occurs. A third, a fourth, and eventually a fifth focus separate from the two initial ParB clusters located close to the cell poles and move toward midcell positions. Cells are aligned with the old pole facing upward; cell lengths are normalized to 1 (*n* = 46). Schemes shown to the left of the graphs illustrate average ParB-eYFP focus positions ± the standard deviations. (C) Time-dependent increase in the number of ParB clusters per cell. Percentages of cells with one to five spots are depicted for each time point (*n* = 30). (D) ParB-eYFP pattern along the cell axis depending on cell length in WT *C. glutamicum*. Automated image analysis of still microscopy images sorted by cell length with high fluorescence intensities displayed in green (*n* >200). (E) Counts of ParB-eYFP spots over time in *C. glutamicum* Δ*parA*. Percentages of cells with 1 to 12 ParB foci were determined for each time point (*n* = 30). (F) Random ParB-eYFP distribution along the longitudinal cell axis in relation to its length in a Δ*parA* mutant strain. Automated analysis of still images with high fluorescence intensities displayed in green (*n* >300). (G) The timing of replication initiation is similar at oriCs of old and young cell poles. Automated image analysis of the *C. glutamicum parB*::*parB-eYFP divIVA*::*divIVA-mCherry* fluorescence pattern sorted by cell length with the old cell pole (high polar DivIVA-mCherry signal level) facing downward. ParB-eYFP (green) and DivIVA-mCherry (red) fluorescence is illustrated in one demograph.

For analysis of chromosome arrangement during one cell cycle, live-cell imaging was performed with *C. glutamicum parB*::*parB-eYFP* (Fig. 3A; see Movie S1). Single cells with two ParB clusters were tracked over one generation time, and focus positions were determined relative to the new pole, revealing distinct origin localization patterns throughout the cell cycle (Fig. 3B). Newborn cells contain two ParB foci stably located close to the cell poles. Newly replicated oriCs segregate from the cell poles toward the midcell position. Often we observed the appearance of a new ParB clusters at either the new or the old pole before a fourth focus separated from the opposite ParB spot. No bias in the timing of origin replication and segregation between old and new cell poles could be detected, as indicated by mean localizations of the third to the fifth focus appearing around the midcell position. A timeline over one generation cycle shows the continuous increase in newly formed segregating oriCs (Fig. 3C). Already after completion of half a cell cycle (~30 min), around half of the cells contained four or five ParB foci; this ratio further increased during growth progression. Uneven ParB focus counts per cell are abundant. Detachment of oriC-ParB complexes from cell poles via chloramphenicol treatment, combined with z-stacking of microscopic images, was used to more accurately determine oriC numbers (see Fig. S2). Up to eight ParB complexes and notably fewer uneven focus numbers were counted per cell than determined with previous experiment settings using untreated cells. These results hint at the absence of asymmetric replication initiation in *C. glutamicum*; hence, uneven ParB-oriC complex numbers are the result of a slightly variable colocalization time of sister oriCs. To corroborate these findings, we used automated analysis of still microscopy images to confirm the spatiotemporal oriC-ParB cluster localization (Fig. 3D). Therefore, we programed a Fiji software plug-in termed Morpholyzer that allows automated cell detection and analysis of fluorescence profiles (see Materials and Methods). High ParB fluorescence intensities were detected close to the poles of all of the cells measured, suggesting stable oriC anchoring at the cell poles. Segregation of sister oriC-ParB clusters toward midcell positions could be detected after around one-fourth of the cell cycle (Fig. 3D). Notably, oriCs stay in the cell half in which they originated. Similar dynamics of oriC localization and segregation patterns have been characterized before for *M. xanthus*, *C. crescentus*, *M. smegmatis*, and *V. cholerae* (33, 62–65). Upon ParA deletion, the time-dependent increase in oriC numbers became less distinct because of large cell length variations directly after cytokinesis (Fig. 3E). As a consequence, multiple oriC-ParB complexes were already present in large newborn cells. Analysis of still images further revealed a disrupted ParB-oriC pattern in *parA* deletion strains compared to the coordinated cellular oriC movement in WT cells (Fig. 3F). Fluorescent foci were detected all along the longitudinal cell axis without clear sites of preference, underlining the crucial role of the partitioning protein ParA in the polar and septal positioning of oriC-ParB clusters in *C. glutamicum*.

10.1128/mBio.00511-17.9 MOVIE S1  Growth of *C. glutamicum parB*::*parB-eYFP* cells in a microfluidic chamber with image acquisition every 5 min for a total of 225 min (AVI format). Download MOVIE S1, AVI file, 0.2 MB. Copyright © 2017 Böhm et al. 2017 Böhm et al. This content is distributed under the terms of the Creative Commons Attribution 4.0 International license.

10.1128/mBio.00511-17.5 FIG S2  Medium dependency of oriC-ParB cluster numbers per cell. (A) Comparison of oriC-ParB complex analysis with (gray bars) and without chloramphenicol treatment of cells combined with z-stacking of microscopic images (black bars). *n* = 200. (B, C) Comparison of growth (B) and distribution of cell lengths and ParB-eYFP cluster numbers (C) (*n* = 200) of the *parB*::*parBeYFP* mutant strain with the use of different carbon sources. Cells were grown in BHI medium (growth rate [µ] = 0.62), BHI+Gluc (µ = 0.49), MMI medium supplemented with glucose (µ = 0.32), and CGXII supplemented with acetate (µ = 0.41) or propionate (µ = 0.13). Experiments were performed in triplicate; error bars display standard deviations. Download FIG S2, TIF file, 0.3 MB. Copyright © 2017 Böhm et al. 2017 Böhm et al. This content is distributed under the terms of the Creative Commons Attribution 4.0 International license.

### Uniform timing of replication initiation at old and young cell poles.

*C. glutamicum* cells grow asymmetrically with unequal rates of peptidoglycan (PG) synthesis at the cell poles. The old cell pole synthesizes more PG than the young pole (66) because of cell cycle-dependent dynamics of DivIVA accumulation (our unpublished data). As *C. glutamicum* DivIVA interacts with the oriC-attached ParB protein (60), the impact of the DivIVA level on chromosome replication or segregation timing was investigated. By using automated analysis of cells coexpressing ParB-eYFP and DivIVA-mCherry, old cell poles were identified by higher DivIVA fluorescence intensities and aligned accordingly. The fluorescence profile shown in Fig. 3G reveals synchronous origin movements toward the newly formed septum from both old and new cell poles, as suggested before by time-lapse analysis (Fig. 3B). Therefore, the timing of chromosome replication seems to be uncoupled from the assembly of the cell wall synthesis machinery despite ParB-DivIVA interaction.

### Longitudinal chromosome arrangement in *C. glutamicum*

**FIG 4:**
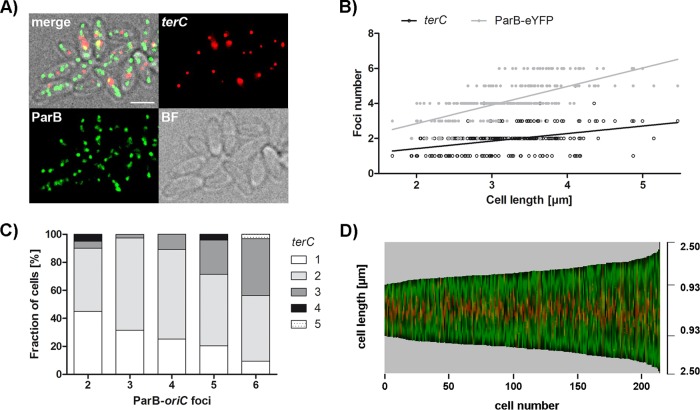
Subcellular terC positioning and cell length-dependent abundance. Microscopic analyses of terC were performed with *C. glutamicum parB*::*parB-eYFP* including a FROS in proximity to terC (*int*::*lacO* pCLTON1PamtR-lacI-CFP) grown in BHI medium. (A) Localization of oriCs and terCs in fast-growing cells. Shown are bright-field (BF), CFP and eYFP fluorescence (terC, ParB), and overlay (merge) images. terCs localize to midcell positions. Scale bar, 2 µm. (B) Numbers of ParB-eYFP and terC foci per cell depending on cell length (*n* = 250). Up to six ParB-oriC and up to five terC complexes were determined per cell. Linear regression lines are shown, terC, *r* = 0.40; ParB-eYFP, *r* = 0.64. (C) Increase in the number of terC foci with the rising number of ParB-eYFP spots per cell. Percentages of cells with one to five terC foci were determined, sorted by oriC complex numbers (*n* = 250). (D) The *C. glutamicum* chromosome is organized longitudinally throughout the cell cycle. Automated image analysis of still microscopy images sorted by cell length reveals the central terC (green) and polar ParB-eYFP (red) fluorescence patterns along the cell axis (*n* >200).

ParB-origin clusters are stably associated with the cell poles, suggesting chromosome termini facing toward the midcell position. To prove an ori-ter-ter-ori chromosome organization in *C. glutamicum*, microscopic analysis of cells harboring fluorescently labeled terminal regions was used. To this end, a fluorescent repressor operator system (FROS) was used in *parB*::*parB-eYFP* cells, with an inducible extrachromosomal copy of *lacI*-*cfp* and *lacO* arrays inserted in proximity to terC. Between one and five terC complexes, with a mean count of 1.94, were observed in brain heart infusion (BHI) medium-grown cells (Fig. 4). Terminus numbers were further quantified at various growth rates, as shown in Fig. S3. Even at the lowest growth rate in propionate-supplemented CGXII medium, up to four foci could be observed. Notably, the frequency distribution of terCs per cell was relatively similar under all of the conditions tested. Likely, numbers were underestimated because of the terC colocalization of several chromosomes. Similar to oriC*-*ParB complexes, terC amounts per cell correlated with cell length. As expected, the number of terminus foci also increased depending on the number of ParB clusters per cell. Figure 4D displays the ParB-eYFP and terC pattern along the longitudinal axis in cells sorted by length. Termini localize to midcell positions throughout the cell cycle, while the opposing origin domains are situated at the cell poles. Interestingly, termini stay in place during the migration of replicated sister oriCs toward them (Fig. 3D shows a spatiotemporal origin pattern). Thus, *C. glutamicum* organizes the chromosomes in a longitudinal fashion with high terC numbers, suggesting more than two chromosomes at a time. This result is in line with unexpected ParB-oriC complex numbers, as shown before.

10.1128/mBio.00511-17.3 FIG S3  Growth rate dependency of terC counts per cell. (A) Growth curves of *C. glutamicum parB*::*parB-eYFP* including a FROS for terC labeling (*int*::*lacO* pCLTON1PamtR-lacI-CFP). Cells were grown in different media, namely, BHI (growth rate [µ] = 0.62), BHI+Gluc (µ = 0.43), MMI medium supplemented with glucose (Gluc) (µ = 0.37), and CGXII supplemented with acetate (Ac) (µ = 0.39) or propionate (Prop) (µ = 0.15). Values were derived from duplicate measurements; error bars display standard deviations. (B) Distribution of terminus numbers per cell depending on the growth rate. One to five terC foci were counted in most of the media used, with the following average terC numbers: BHI, 1.94; BHI Gluc, 1.68; MMI Gluc, 1.66; CGXII Ac, 1.66; CGXII Prop, 1.74 (*n* = 250). (C) Representative cells grown at defined rates. Shown are bright-field (BF), terC FROS and ParB fluorescence (terC, ParB), and complement images (merge). Download FIG S3, TIF file, 1 MB. Copyright © 2017 Böhm et al. 2017 Böhm et al. This content is distributed under the terms of the Creative Commons Attribution 4.0 International license.

### Replisome tracking reveals multiple replication forks and variable origin cohesion times.

**FIG 5:**
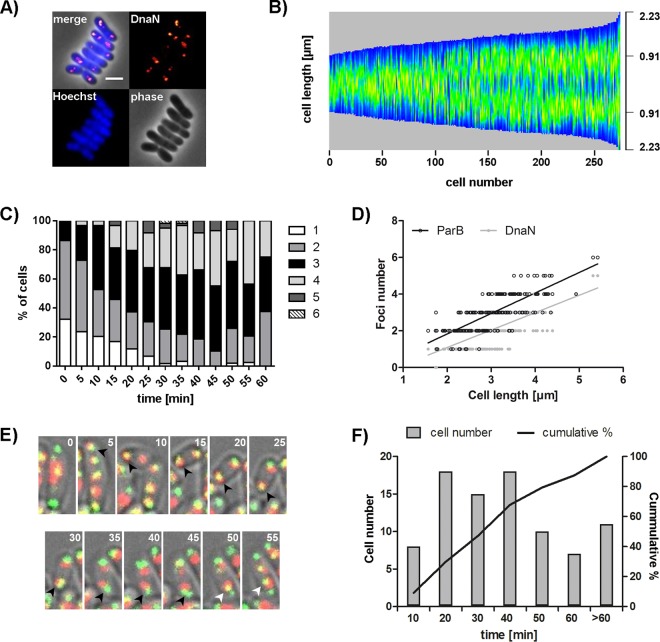
Dynamic localization of multiple replisomes in *C. glutamicum*. (A) Localization of replisomes in *dnaN*::*dnaN-mCherry* mutant cells. Shown are an overlay (merge) of DnaN fluorescence (red) and DNA stained with Hoechst (blue) with a phase-contrast image and separate channels (DnaN, Hoechst, and phase). Scale bar, 2 µm. (B) Timing of replication along the cell axis. Automated analysis of still images with DnaN-mCherry fluorescence. Cells are sorted by length, and DnaN-mCherry fluorescence is shown as a heat map (blue to orange) (*n* >250). (C) Replisome numbers per cell vary within one cell cycle. Percentages of cells with one to six DnaN spots were determined for each time point (*n* = 59). (D) ParB and DnaN focus numbers in relation to cell length in *C. glutamicum parB*::*parB-eYFP dnaN*::*dnaN-mCherry* (*n* = 200). Linear regression lines are shown. ParB-eYFP/DnaN-mCherry, *r* = 0.65. (E) Time frames of replication initiation until segregation of sister oriCs. Shown is a time series showing the movement of ParB-eYFP and DnaN-mCherry foci (green and red, overlay in yellow) in *parB*::*parB-eYFP dnaN*::*dnaN-mCherry* mutant cells. Images were taken at 5-min intervals, as indicated in the top right corners. At 5 min, a replisome forms at the polar oriC (black arrowheads); sister oriCs separate at 50 min (white arrowheads). (F) Variable cohesion periods of sister oriCs. Shown is the distribution of oriC colocalization times analyzed by time-lapse microscopy together with the cumulative skew of sample data (*n* = 88).

*In vivo* characterization of replisome dynamics was carried out with a reporter strain in which the native locus of helicase sliding-clamp *dnaN* was replaced with a *dnaN*-*mCherry* fusion construct (Fig. 5A). Cell length distribution and growth of the DnaN-mCherry-expressing strain resemble the WT situation (see Fig. S1), and the presence of full-length DnaN-mCherry protein could be confirmed via Western blot analysis (see Fig. S1), suggesting that the localization patterns are not due to free fluorophores or degraded protein. To track the pattern of replication timing, automated analysis of fluorescence microscopy images of cells grown in BHI medium was used (Fig. 5B). In growing cells, high DnaN-mCherry signal levels were observed in a wide range around the midcell position. At the end of the first third of the generation time, a fluent transition of replication termination around the midcell position toward the formation of newly formed replication hubs in cell quarter positions took place. This large-scale microscopy analysis clearly indicates that new rounds of replication initiation cannot be temporally separated from the previous ones. C periods follow each other at short intervals or might even overlap under fast-growth conditions. Notably, single-cell analysis can show that replisomes are formed at polar or septal oriC-ParB complexes and gradually move away from the origins toward the midcell position (see Fig. S4). Such a DnaN fluorescence pattern is not immediately obvious in demographs, presumably because of variable timing of replication initiation between cells of similar sizes. Further, we questioned whether replication forks translocate actively to the midcell position or whether the observed movement is an effect of polar cell elongation in *C. glutamicum*. To this end, cellular DnaN movement was analyzed via time-lapse microscopy (see Fig. S5D). Subtraction of half the cell length increase from the distance covered by a replisome in a time frame from DnaN focus appearance until cell division yielded an average distance of zero. The possibility cannot be excluded that translocation of polar replisomes toward the midcell position is a passive process solely caused by polar cell elongation in *C. glutamicum*. The movement of replication forks observed by live-cell imaging appeared to be highly dynamic (see Movie S2). During live-cell imaging, a progressive increase in DnaN focus numbers over one generation time was observed (Fig. 5C). Initially, two replication forks were counted for most of the cells; the number further increased to up to six foci per cell throughout the cell cycle, indicating three or more simultaneous replication events per cell. Notably, replisome splitting and merging occur, and because of high DnaN numbers, focus overlays cannot be excluded. To further analyze replication initiation and progression depending on origin localization, a dual-reporter strain expressing ParB-eYFP and DnaN-mCherry was constructed. The WT-like growth and cell lengths of this strain were confirmed (see Fig. S1). The dependence of ParB-eYFP and DnaN-mCherry focus numbers on cell length is shown in Fig. 5D. On average, fewer DnaN than ParB spots were counted per cell, as indicated by regression lines. However, the replication fork number could have been underestimated because of frequent merging of forks initiated from the same origin. These results reveal simultaneous replication events of several chromosome equivalents per cell. Furthermore, a moderate correlation between the numbers of oriC-ParB clusters and replication forks per cell could be determined. By using live-cell imaging, cohesion periods of sister origins were analyzed, which are defined as the time between the formation of a new DnaN-mCherry spot in colocalization with an oriC-ParB cluster and the subsequent splitting of the latter into two distinct fluorescent signals (Fig. 5E). Time frames between replication initiation and sister origin segregation are illustrated in Fig. 5F. Since this approach is limited by the resolution of microscopic analysis, ParB cohesion could have been, on average, overestimated. However, cohesion periods appear to be highly variable. Time spans of 5 to 80 min were measured, with an average period of 36 min. The mean interval length is comparable to the 40-min origin colocalization time in fast-growing *E. coli* cells determined before (67). Further, a tight regulation of their origin cohesion periods might be absent from this organism. Replication initiations in the mother generation could frequently be observed with origin splitting only in subsequent generations, as exemplified in Fig. 5F. Notably, new rounds of chromosome replication were initiated at polar, as well as at midcell-positioned, origins (see Fig. S4).

10.1128/mBio.00511-17.4 FIG S4  Single-cell analysis of replisome dynamics. Replication cycle accounting for gradual replisome movement toward the midcell position. Shown are time series exemplifying the movement of replisomes relative to oriC-ParB complexes (green and red, overlay in yellow) in a strain encoding ParB-eYFP and DnaN-mCherry as allelic replacements of the native gene products. Images were taken at 5-min intervals (bottom right corner). At 5 min, polar replisomes assemble in a predivisional cell (white arrowheads), where newly replicated sister oriCs colocalize for up to 40 min; replisomes gradually move away from polar oriCs. Prior to replication termination, a new round of replication is initiated at 60 min (black arrowhead). Sister replisomes merge in one fluorescent spot but frequently split (50 and 55 min, see cartoon below images). Scale bar, 2 μm. Download FIG S4, TIF file, 2.4 MB. Copyright © 2017 Böhm et al. 2017 Böhm et al. This content is distributed under the terms of the Creative Commons Attribution 4.0 International license.

10.1128/mBio.00511-17.6 FIG S5  Growth rate dependency of replisome numbers per cell. Cells were grown exponentially in BHI medium (BHI), BHI+Gluc, or MMI medium supplemented with glucose (MMI Gluc). (A) Images exemplify the localization of replisomes in *dnaN*::*dnaN-mCherry* mutant cells. Shown are phase-contrast images (phase), mCherry fluorescence (DnaN), and overlays of both channels (merge). Scale bar, 2 µm. (B) DnaN-mCherry counts per cell depending on the growth medium. Between zero and eight foci were determined per cell (*n* = 300). (C) Relationship between the replisome number and cell length. Linear regression lines are shown for cells grown in distinct media as follows: BHI, *r* = 0.54; BHI+Gluc, *r* = 0.55; MMI Gluc, *r* = 0.42 (*n* = 300). (D) Replisome translocation from the poles toward the midcell position likely arises because of polar cell growth. Shown is the difference between the cell length increase divided by two and the distance covered by the replisome from DnaN focus appearance until cell division. Length = 0.014 μm, standard deviation = 0.242 μm (*n* = 52). Download FIG S5, TIF file, 1.4 MB. Copyright © 2017 Böhm et al. 2017 Böhm et al. This content is distributed under the terms of the Creative Commons Attribution 4.0 International license.

10.1128/mBio.00511-17.10 MOVIE S2  Growth of *C. glutamicum dnaN*::*dnaN-mCherry* cells in a microfluidic chamber with image acquisition every 5 min for a total of 230 min (AVI format). Download MOVIE S2, AVI file, 0.3 MB. Copyright © 2017 Böhm et al. 2017 Böhm et al. This content is distributed under the terms of the Creative Commons Attribution 4.0 International license.

### Overlapping replication periods allow fast growth.

**FIG 6:**
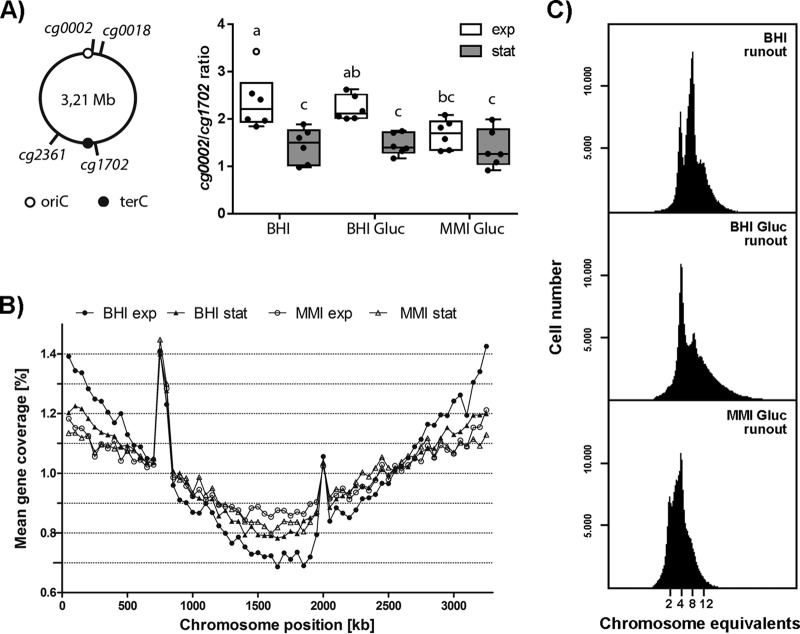
Timing of DNA replication initiation and determination of oriC numbers per cell. (A) Marker frequency analysis of oriC and terminus regions. (Left) Schematic representation of the chromosomal positions of the oriC- and terC-proximal marker genes used. (Right) oriC-to-terminus ratios of the WT strain grown under different conditions were determined by frequency analysis of markers *cg0002* and *cg1702* (see Fig. S6B for *cg0018*/*cg2361* ratios). Cells were grown in BHI medium, BHI+Gluc, or MMI supplemented with glucose. Samples were taken in the exponential (exp, white boxes) and stationary (stat, gray boxes) growth phases. Results are shown as box plots with medians indicated as solid lines and whiskers of 1.5 times the interquartile range (*n* = 6); outliers are depicted as open circles. ANOVA yielded significant variation among growth phase (*F*^1,30^ = 28.00, *P* < 0.0001) and medium (*F*^2,30^ = 3.43, *P* < 0.05) conditions. Letters indicate significant differences between data sets determined by *post-hoc* Bonferroni analysis at *P* < 0.05. (B) Whole-genome sequencing. Genomic DNA of WT *C. glutamicum* grown in BHI or MMI medium supplemented with glucose was isolated in the exponential and stationary growth phases. Data were analyzed by Illumina MiSeq shotgun sequencing and mapped to the *C. glutamicum* ATCC 13032 genome sequence (GenBank accession no. BX927147.1). Data are displayed as the mean gene coverage of each 50-kb sliding window as a percentage of the total mean coverage per sample. Note that the RES167 strain used in this study lacks the phage island (*cg1981*-*cg2034*) and harbors an *ISCg14*-mediated 5-fold tandem amplification of the *tus* locus (peaks at approximately 750- and 2,000-kb positions); both loci were excluded from data analysis. Stable replication progression is evidenced by the frequency of genes between the oriC (located at 0 kb) and terminus regions (at approximately 1.6 Mb). (C) Numbers of chromosomes per cell determined by flow cytometry after replication runout in BHI medium, BHI+Gluc, or MMI medium supplemented with glucose. Depending on the growth conditions, between 2 and 12 chromosomes were detected.

The observation of multiple DnaN and ParB foci suggests the possibility that *C. glutamicum* is able to initiate new replication rounds before the ongoing replication is finished. To test this hypothesis, we used marker frequency analysis to investigate the growth rate-dependent replication patterns of *C. glutamicum*. We determined the oriC/terC ratios of cells grown in three different media (BHI medium, BHI medium with 4% glucose [BHI+Gluc], and minimal medium with 4% glucose [MMI]), allowing fast, intermediate, and slow growth (see Fig. S6A). Data from quantitative PCR (qPCR) experiments using markers of origin- and terminus-proximal regions prove the growth rate dependency of oriC/terC ratios (Fig. 6A). As a control, we analyzed *B. subtilis*, an organism with clear multifork replication. Exponentially grown *B. subtilis* cells have oriC/terC ratios considerably above 2 (see Fig. S6C). Analysis of exponentially growing *C. glutamicum* cells cultured in BHI medium and BHI+Gluc yielded mean oriC/terC ratios of 2.4 and 2.2, indicating an overlap of replication periods. Under slow-growth conditions in MMI medium, the mean oriC*/*terC ratio of 1.7 did not significantly differ from values obtained with cells in the stationary growth phase. Upon antibiotic treatment leading to replication runouts (by inhibiting replication initiation yielding fully replicated chromosomes), the ratios dropped to values close to 1 (data not shown). These results were further supported by whole-genome sequencing of cells grown in BHI and MMI media (Fig. 6B). Sequencing coverages revealed a symmetric progression of replication forks on both arms of the chromosome under all of the conditions tested. During exponential growth in BHI medium, the mean gene coverage in origin regions was around 2.1 times that measured in terminus-proximal regions. The oriC/terC ratio dropped to 1.5 for log-phase cells grown in MMI medium, as well as for cells in the stationary growth phase. These results hint at a fraction of cells that did not complete replication during stationary growth. Likewise, active replication forks were observed in around 24% of the stationary-phase *M. smegmatis* cells (65). We also analyzed oriC/terC ratios in the Δ*parA* mutant strain via qPCR (see Fig. S6D). These data do not hint at replication overinitiation in the strain lacking *parA* in comparison to that in the WT, supporting our notion, mentioned above, that higher oriC numbers in the Δ*parA* mutant strain are likely caused by a loss of oriC-ParB complex cohesion or reduced tethering to cell poles but not by overinitiation.

10.1128/mBio.00511-17.7 FIG S6  Examination of replication initiation. (A) Growth curves of WT *C. glutamicum* grown in BHI medium, BHI+Gluc, or MMI medium supplemented with glucose. Genomic DNA was isolated in the exponential and stationary growth phases and used in marker frequency experiments and for whole-genome sequencing. Values were derived from triplicate measurements; standard deviations are indicated in all graphs. For growth rates, see Table 1. (B) oriC-to-terminus ratios determined via marker frequency analysis of chromosomal loci *cg0018* and *cg2361*; cf. Fig. 5A. ANOVA revealed an effect of the growth phase (*F*^1,30^ = 13.41, *P* < 0.001), of the medium used (*F*^2,30^ = 9.77, *P* < 0.001), and a significant interaction of the growth phase and the medium used (*F*^2,30^ = 7.17, *P* < 0.01). (C) Marker frequency analysis of *B. subtilis* 168 genomic DNA (marker in intergenic oriC and terC regions) isolated from cells grown in LB at time points of exponential-phase growth (oriC/terC ratio of >10) to stationary-phase growth (oriC/terC ratio of <2). (D) No overinitiation in the Δ*parA* mutant in comparison with that in WT cells was determined by qPCR of oriC/terC ratios. Cells were grown in BHI medium, and samples were taken in the exponential (white bars) and stationary (gray bars) growth phases. Shown are the mean and standard deviation of triplicate measurements, with WT values set to 1 and Δ*parA* mutant data normalized accordingly. Download FIG S6, TIF file, 0.2 MB. Copyright © 2017 Böhm et al. 2017 Böhm et al. This content is distributed under the terms of the Creative Commons Attribution 4.0 International license.

Marker frequency analysis and genome sequencing results point to growth rate-dependent replication cycles in *C. glutamicum* cells, where the timing of a new initiation precedes termination of the previous replication event to enable fast growth.

### *C. glutamicum* cells contain multiple chromosome equivalents at various growth rates.

As shown by marker frequency analysis, oriC/terC ratios are considerably higher in fast-growing than in slow-growing cells. To more precisely verify the DNA content per cell, flow cytometry was used. To this end, replication runouts of WT cells cultured at various growth rates were performed and nucleoids were fluorescently stained with SYBR Green I dye. DNA histograms show the numbers of fully replicated chromosomes, which equal the cellular origin numbers at the time point of antibiotic treatment (Fig. 6C). Absolute DNA content was assigned according to an internal standard (see Fig. S7A and B). The DNA content distributions of cells at the exponential or stationary growth phase not subjected to antibiotic treatment are shown in Fig. S7C for all of the conditions tested. Fast- and intermediate-growth conditions gave rise to mainly four and eight chromosomes per cell, and a smaller fraction of cells contained 10 and 12 chromosomes. Slow-growth conditions yielded mainly cells containing either two or four chromosomes and a small subpopulation containing eight chromosomes. Our data result in average chromosome numbers per cell of 5.90, 5.17, and 3.85, from the highest growth rate to the lowest, respectively. Strikingly, the number of chromosome equivalents determined by flow cytometry is considerably higher than expected from marker frequency results, and monoploid cell fractions were absent under all of the growth conditions tested in the exponential phase. Flow cytometry results are further supported by fluorescence microscopy analysis of WT *parB*::*parB-eYFP* cells cultivated at several different growth rates. Cells with fewer than two ParB foci per cell were almost absent under all of the condition tested. In addition, ParB-origin cluster numbers could possibly be underestimated because of potential origin cohesion (see Fig. S2). Replisome numbers per cell also depend on growth rates (Fig. S5), further supporting our results. Similar to oriC-ParB complexes, replisome numbers correlate with cell length for all of the conditions tested and fast growth in BHI medium or BHI+Gluc allows for one to six DnaN foci per cell, whereas zero to four DnaN spots were counted in MMI-grown cells. Thus, we conclude that *C. glutamicum* is at least diploid, with two chromosomes attached to the cell poles via the centromeric oriC-ParB nucleoprotein complex. Overinitiation of DNA replication leads to multiforked chromosomes under fast-growth conditions.

10.1128/mBio.00511-17.8 FIG S7  Calibration of DNA measurements. *B. subtilis* cells with known DNA content served as the internal standard in flow cytometry measurements after the application SPAAC cell wall staining with carboxyrhodamine (CR). The standard was included in samples of *C. glutamicum* cells grown at different rates; DNA was stained with Hoechst dye. (A) Flow cytometry sample of *B. subtilis* and *C. glutamicum* cells. Shown is the overlay (merge) of carboxyrhodamine (green) and Hoechst (blue) fluorescence with the phase-contrast image and individual channels (CR, phase, Hoechst). Scale bar, 2 µm. (B) Gating strategy exemplified on a sample containing BHI medium-grown *C. glutamicum* cells. (Left) *C. glutamicum* and *B. subtilis* subpopulations were identified in density plots of the carboxyrhodamine fluorescence intensity (CR FI, arbitrary units) channel versus the Hoechst fluorescence intensity (Hoechst FI, arbitrary units) channel according to their green fluorescence intensity. (Right) Determination of *C. glutamicum* chromosome numbers (black) according to the *B. subtilis* standard (red) in histograms versus DNA content. (C) Distributions of DNA content in WT *C. glutamicum*. Cells were grown in BHI medium (left), BHI+Gluc (middle), or MMI medium supplemented with glucose (MMI Gluc, right). Samples of exponential-phase cultures were taken prior to chloramphenicol treatment (exp.), after 4 h of incubation with (+Cm) or without chloramphenicol (in an already stationary state [stat.]). Cell fixation, staining, and flow cytometry were carried out subsequently. Download FIG S7, TIF file, 0.3 MB. Copyright © 2017 Böhm et al. 2017 Böhm et al. This content is distributed under the terms of the Creative Commons Attribution 4.0 International license.

### Growth rate-dependent cell cycle models.

Cell cycle parameters derived from marker frequency and flow cytometry analyses (Table 1) allowed us to formulate complete cell cycle models for *C. glutamicum* under different growth conditions. A C period of 78 min was determined for cells grown in BHI medium, whereas intermediate and low growth rates are associated with slightly longer replication periods of 96 and 97 min. These values result in a DNA replication speed of around 340 bases/s, which is in the range of replication speeds reported for *C. crescentus*, *M. xanthus*, and *M. smegmatis* (Table 2 and references therein). D-period equations reported before (68) yielded time intervals longer than the doubling time for each of the three growth conditions analyzed, indicating the presence of two fully replicated chromosomes per newborn cell. Since we define the D period as the time interval from replication termination that took place in the current generation until subsequent cell division, D-period calculation was adapted to a diploid organism (see Materials and Methods). Those time frames remained relatively unaltered, with 20, 18, and 26 min from high to low growth rates, respectively.

**TABLE 1:** Overview of *C. glutamicum* cell cycle parameters at distinct growth rates

Growth medium	μ^a^ (1/h)	*T*_d_^b^ (min)	oriC/terC ratio	No. of oriCs/cell	C period (min)	D period (min)
BHI	0.66	63	2.36 ± 0.54	5.90 ± 0.03	78	20
BHI+Gluc	0.50	83	2.23 ± 0.21	5.17 ± 0.02	96	18
MMI+Gluc	0.32	130	1.68 ± 0.28	3.85 ± 0.16	97	26

a μ, growth rate.

b *T*_d_, doubling time.

**TABLE 2:** Speed of DNA replication forks summarized for different model organisms

Organism	Genome size (Mb)	C period (min)	Replication speed (bases/s)	Reference(s)
*Corynebacterium glutamicum*	3.21	78	340	This work
*Mycobacterium tuberculosis*	4.42	660	50	87
*Mycobacterium smegmatis*	6.99	140	400	64, 65
*Myxococcus xanthus*	9.14	200	380	62, 88
*Caulobacter crescentus*	4.02	95	350	89
*Vibrio cholerae^a^*	2.96	30–50	490–820	17, 90
*Bacillus subtilis*	4.22	50–60	600–700	68, 91
*Escherichia coli*	4.64	40–200	200–1,000	15, 68, 92–94

a Shown are parameters for chromosome I only.

**FIG 7:**
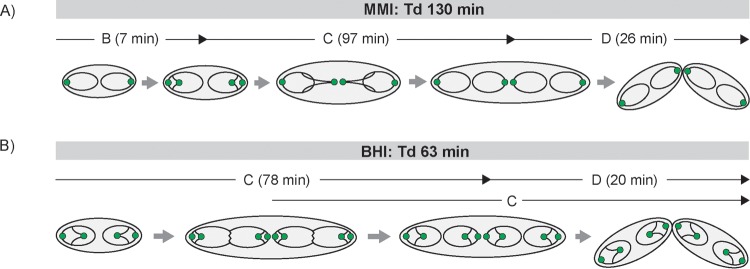
Spatiotemporal chromosome organization of *C. glutamicum*. Chromosomes are depicted as black lines with oriCs as green circles. In newborn cells, two initial oriCs are located close to the poles. Upon initiation of a new round of replication, sister oriCs segregate nonsynchronously from polar ParB-eYFP clusters and move toward the midcell position, where a new septum is formed. Notably, stages with single chromosomes per cell are absent. (A) Cell cycle of slow-growing cells in MMI medium. A short B period is followed by C and D periods; replication takes place within one generation. (B) Chromosome organization during fast growth in BHI medium. Multifork replication allows for short doubling times (Tds), with a second round of replication starting after the first half of the cell cycle, around 15 min before the previous one terminates.

Growth rate-dependent cell cycle modes are illustrated for fast- and slow-growth conditions (Fig. 7). Newborn cells localize their origins in two clusters at polar positions, with chromosomes being arranged longitudinally. During cell cycle progression, sister oriC-ParB complexes segregate and move toward septal positions, whereby up to four ParB foci per cell in MMI and up to six ParB foci per cell in BHI medium could be detected microscopically. Overlapping C periods allow for short doubling times of as little as 63 min, with a new round of replication initiating 28 min after cell division and the ongoing replication event terminating 15 min later. Cells with long doubling times of 130 min possess a short time interval of 7 min between cell division and replication initiation (B period) and fully replicate both chromosome copies once per generation. In both the fast- and the slow-growth models, cells are diploid, since newly replicated sister chromosomes will only be separated by cell division in the daughter generation. Thus, the cell cycle models suggested here describe overlapping replication cycles in combination with two sets of chromosomes in *C. glutamicum*.

## DISCUSSION

The bacterial cell cycle has been analyzed in few model organisms in the past. A hallmark of fast-growing species such as *E. coli* and *B. subtilis* is the initiation of new rounds of DNA replication prior to replication termination and cytokinesis (15, 16). This process has been termed multifork replication. In slow-growing species or species with asymmetric division such as *C. crescentus* and *M. smegmatis*, C periods do not overlap (13, 14). The increasing knowledge of bacterial cell biology has made it clear that even fundamental cell processes such as cytokinesis and DNA replication might be organized and regulated in a far higher diversity than initially thought (69, 70). We have therefore analyzed the cell cycle of *C. glutamicum* under various growth conditions and at different growth rates. *C. glutamicum* emerges as a model organism for apical cell growth that is characteristic of members of the order *Actinomycetales* (61).

Analysis of ParB foci in growing *C. glutamicum* cells revealed that under all of the culture conditions tested, a ParB focus is stably attached to each pole and terCs face toward the midcell position, suggesting an ori-ter-ter-ori orientation of the chromosomes (Fig. 2 to 4). Interestingly, newly replicated origins segregate to the midcell position, where they remain until cytokinesis is completed and they stay tethered to the newly forming cell poles (Fig. 3). Until now, it has been unclear by which molecular mechanism the placement of ParB and the chromosomal origin is directed. Earlier work has shown that ParB and the cell division protein FtsZ interact (51). Interaction of ParB with the divisome might be a plausible explanation for the observed localization pattern. Deletion of ParA completely abolishes the directed ParB segregation, confirming earlier results obtained by our group (51). Thus, unlike in other species with polar origin localization, such as *C. crescentus* and *V. cholerae*, the newly replicated origin does not migrate to the opposite pole (32, 71). This mode of segregation in *C. glutamicum* is compatible with the observation that both cell poles are constantly occupied with a ParB-oriC complex, suggesting that even newborn cells contain at least two chromosomes and hence are diploid. Because of the variable cohesion times of sister chromatids (Fig. 5C), the number of ParB foci does not necessarily reflect the number of origins and may lead to an underestimation of origins. Since cell elongation and cytokinesis are spatially less well regulated in *C. glutamicum* than in *E. coli* or *B. subtilis*, cellular chromosome content can be variable, explaining small cell fractions with origin or terminus numbers that deviate from the model (Fig. 4; see Fig. S3). The existence of several chromosomes is in line with the observation of multiple replication forks. During replication, two or more replication forks can be visualized, as judged by fluorescently labeled sliding-clamp DnaN (Fig. 5). The localization of origins in *C. glutamicum* is therefore different from that in the closely related species *M. smegmatis*. For *M. smegmatis*, a replication factory model has been proposed in which a centrally localized origin is replicated and the newly replicated origins are segregated toward the cell pole while the replisome remains in the cell center (64). In contrast, we show by time-lapse analysis that replication forks originate close to the cell pole and migrate toward the midcell position, possibly because of apical cell elongation in *C. glutamicum* (Fig. 5E; see Fig. S4). However, further proof is needed to distinguish replisome migration along its template DNA being pulled along with the growing pole from a static replication factory, as proposed for *B. subtilis* (72). We also observed that both origins initiate replication around the same time. Further support for diploidy stems from flow cytometry data (Fig. 6). In replication runout experiments, cells contain a minimum of 2 and up to 12 chromosomes, which is a clear indication of multiple initiations under fast-growth conditions (growth rates of >0.6 h^−1^). Although the presence of multiple chromosomes per cell has been suggested before (73), only single-cell analysis unambiguously supports the diploidy of *C. glutamicum* cells. The simultaneous presence of two polar chromosomes is in stark contrast to findings recently reported for *Mycobacterium* species (64, 65). Corynebacteria and mycobacteria are closely related, and hence, it comes as a surprise that they might differ in the cellular organization of their chromosomes. The constant diploidy of *C. glutamicum* could be a consequence of its environmental lifestyle. Many soil bacteria have elaborated sophisticated methods to counteract various environmental stresses such as desiccation, nutrient shortage, or exposure to DNA-damaging agents. In fact, nucleic acids are prominent targets for desiccation-induced damage (74). Cells carrying two or more chromosome equivalents will increase the chance for correct DNA repair based on homologous recombination. In line with this hypothesis, survival rates of coryneform bacteria are known to be high under various stresses, including desiccation (75–77). Analyses of long-term preservations of microbial ecosystems in permafrost demonstrate that corynebacteria dominate older sediments (77).

In summary, we have provided detailed analyses of the cell cycle of *C. glutamicum* at different growth rates (Fig. 7). Data presented here point to a unique and previously undescribed mode of cell cycle regulation with two pole-attached chromosomes that simultaneously initiate replication. Under fast-growth conditions, new rounds of replication can be initiated before the previous round is complete, similar to multifork replication. In contrast to other bacteria with pole-oriented chromosomes, such as chromosome I from *V. cholerae* or *C. crescentus*, *C. glutamicum* cells contain two copies of the chromosomes and segregate the newly replicated origins only to the midcell position. Elucidation of the corynebacterial cell cycle is important for a full understanding of the growth behavior and homologous recombination of this medically and industrially relevant genus.

## MATERIALS AND METHODS

### Oligonucleotides, plasmids, and bacterial strains.

All of the primers, plasmids, and bacterial strains used in this study are listed in Tables S1 and S2 in Text S1.

10.1128/mBio.00511-17.1 TEXT S1  Table S1 describes the oligonucleotides used in this study, and Table S2 describes the bacterial strains and plasmids used in this study. Download TEXT S1, DOCX file, 0.1 MB. Copyright © 2017 Böhm et al. 2017 Böhm et al. This content is distributed under the terms of the Creative Commons Attribution 4.0 International license.

### Strain construction.

Integration plasmids were constructed with 500-bp homologous regions upstream and downstream of the 3′ end of the gene to be labeled with a fluorophore sequence in between. For plasmids pK19mobsacB-parB-eYFP and pK19mobsacB-parB-mCherry2, the upstream and downstream fragments were PCR amplified from the *C. glutamicum* genome with primer pairs ParB-Hind-up-F/ParB-Sal-up-R and ParB-XbaI-D-F/ParB-Bam-D-R. Enhanced yellow fluorescent protein (eYFP) or mCherry2 sequences were amplified with primer pairs eYFP-SalI-F/eYFP-XbaI-R and mCherry2-SalI-F/mCherry2-XbaI-R. To construct pK19mobsacB-DnaN-mCherry, upstream and downstream homologous regions were amplified via primer pairs DnaN-Hind-up-F/DnaN-SphI-up-R and DnaN-XbaI-D-F/DnaN-BamHI-D-R. The mCherry sequence was amplified with primer pair mCherry-SalI-F/mCherry-XbaI-R. The resulting PCR fragments were digested with the respective restriction enzymes and consecutively ligated into pK19mobsacB vectors. Plasmid cloning was performed with *E. coli* DH5α. *C. glutamicum* was transformed via electroporation and selected for integration of the fluorophore as described before (78). To confirm the allelic replacements, *parB*::*parB-eYFP* and *parB*::*parB-mCherry2* colony PCR was carried out with primers ParB-N-ter-SalI-F and ParB-PstI-800D-R, and for the allelic replacement *dnaN*::*dnaN-mCherry*, primers DnaN-N-ter-F and DnaN-Bam-700D-R were used. For terminus tracking, a FROS was constructed in *C. glutamicum parB*::*parB-eYFP*. To this end, the terminus-proximal *int* gene (*cg1752*) was replaced with a *lacO* array (~120 operator copies) cut out of pLAU43 with XbaI and XmaI. Upstream and downstream homologous flanking sequences of *int* were PCR amplified with primer pairs Int-HindIII-up-F/Int-PstI-up-R and Int-EcoRI-D-F/Int-NheI-D-R, and the resulting PCR fragments were digested with restriction enzymes as indicated in Table S1 in Text S1. All restricted fragments were subsequently ligated into plasmid pK19mobsacB, yielding pK19msB-int::lacO, which was cloned into *C. glutamicum parB*::*parB-eYFP*. Gene replacement was confirmed by colony PCR with primer pairs Int-700up-F and Int-700D-R. For pCLTON1PamtR-*lacI-CFP* construction, the lacI-cyan fluorescent protein (CFP) sequence of pLAU53 was PCR amplified with LacI-SalI-F/CFP-KpnI-R and digested with the respective restriction enzymes. Subcloning was performed with pEKEx2, and after restriction digestion with PstI and KpnI, the fragment was ligated into pCLTON1PamtR. This plasmid was cloned into *C. glutamicum parB*::*parB-eYFP int*::*lacO*.

### Growth conditions and media.

*B. subtilis* and *E. coli* cells were grown at 37°C in lysogeny broth (LB) supplemented with 25 µg/ml kanamycin when appropriate. *C. glutamicum* cells were grown at 30°C in BHI medium (Oxoid), BHI+Gluc, MMI medium (79) supplemented with 4% glucose, or CGXII medium (80) supplemented with 120 mM acetate or 100 mM propionate, as indicated. For growth experiments in BHI+Gluc, MMI, or CGXII medium, cells were inoculated into BHI medium and diluted in growth medium overnight for precultivation. The next morning, cultures were diluted to an optical density at 600 nm (OD_600_) of 1. Growth in BHI medium preceded an overnight inoculation step; cultures were rediluted to an OD_600_ of 0.5 the next morning. Extrachromosomal *lacI-cfp* expression was induced with 0.15 µg/ml tetracycline. For replication runouts, exponentially growing *C. glutamicum* or *B. subtilis* cells were treated with 25 or 200 µg/ml chloramphenicol for 4+ h.

### Fluorescence microscopy and live-cell imaging.

Fluorescence microscopy was carried out with an Axio-Imager M1 fluorescence microscope (Carl Zeiss, Inc.) with an EC Plan Neofluar 100×/1.3 oil Ph3 objective and a 2.5× Optovar for automated image analysis. Filter sets 46 HE YFP (excitation [EX] band pass [BP] 500/25 nm; beam splitter [BS] 515 nm; emission [EM] BP 535/30 nm) and 43 HE Cy3 shift free (EX BP 550/25 nm; BS 570 nm; EM BP 605/70 nm) were used for fluorescence detection of eYFP and mCherry or mCherry 2 protein fusions. DNA was stained with 1 µg/ml Hoechst 33342 (Thermo Scientific). For live-cell imaging, cells in the exponential growth phase were rediluted to an OD_600_ of 0.01 in BHI medium and loaded into a microfluidic chamber (B04A CellASIC; ONIX) at 8 lb/in^2^ for 10 s; for nutrient supply, 0.75 lb/in^2^ was used. Time-lapse microscopy was performed with a Delta Vision Elite microscope (GE Healthcare, Applied Precision) with a standard four-color InSightSSI module and an environmental chamber heated to 30°C. Images were taken with a 100×/1.4 oil PSF U-Plan S-Apo objective and mCherry (EX BP wavelengths, 575 to 25 nm; EM BP wavelengths, 625 to 45 nm)- or YFP (EX BP wavelengths, 513 to 17 nm; EM BP wavelengths, 548 to 22 nm)-specific filter sets were used for fluorescence detection (50% transmission, 0.3-s exposure). Images were taken at 5-min intervals. Stacks of six z-planes were taken with 0.2-µm section widths for terC and ParB-eYFP focus counting as indicated. For data analysis, FIJI software (81) was used; cell length measurements were acquired manually.

### Marker frequency analysis.

Genomic DNA was isolated from *C. glutamicum* or *B. subtilis* cells in the exponential or stationary growth phase. DNA proximal to the origin or terminus (see Results) was amplified by qPCR with 2× qPCR Mastermix (KAPA SYBRfast; Peqlab) in accordance with the manufacturer’s protocol. Samples were set to 10 µl containing 200 nM primer and 1.5 ng of DNA. Primer efficiency was tested for all of the oligonucleotides used (see Table S1 in Text S1) and estimated by calibration dilution curves and slope calculation (82). Each experiment was performed in technical triplicate on an iQ5 multicolor real-time PCR detection system (Bio-Rad), and threshold cycle (*C*_*T*_) values were determined with the Bio-Rad iQ5 software version 2.1. Results were analyzed via the 2^−Δ*CT*^ method (83). DNA replication runouts (see the description of growth conditions and media above) yielding an oriC/terC ratio of 1 served as a reference.

### Flow cytometry analysis.

Culture samples were fixed 1:9 (vol/vol) in 70% ethanol and stored at 4°C until use. Cells were pelleted at 5,000 rpm for 5 min and washed once in phosphate-buffered saline (PBS). The DNA staining procedure used was adapted from a protocol described before (84). Samples were preheated to 37°C and stained with SYBR Green I (Invitrogen) at a final dilution of 1:10,000 for 15 min and consequently diluted in PBS. Flow cytometry analysis was performed with a BD Accuri C6 (BD Biosciences) with a 488-nm blue laser. Measurements were conducted at a flow rate of 10 µl/min with an acquisition threshold set to 700 on FL1-H and a rate of <5,000 events/s. At least 200,000 events per sample were collected. Data were analyzed by plotting samples as histograms versus the green channel (FL1-A, EM BP, 533/30 nm) on a log scale. All experiments were performed in biological triplicate.

To calibrate the DNA measurements of different growth conditions, *B. subtilis* cells were used as an internal standard. A replication runout of *B. subtilis* cells grown in LB gave rise to cells with mainly four or eight fully replicated chromosomes (68). Prior to ethanol fixation, the cell wall was stained via strain-promoted alkyne-azide cycloaddition (SPAAC). In short, 5 mM 3-azido-d-alanine (Baseclick GmbH), which is incorporated into the cell wall, was added to the culture during the time of replication runout. Cells were washed in PBS, incubated with 10 µM dibenzocyclooctyl-(polyethylene glycol) 4−5/6-carboxyrhodamine 110 (Jena Bioscience) at 30°C for 20 min in the dark, and subsequently washed three times in PBS--0.1% Tween 80. This standard was included with *C. glutamicum* cells during incubation with 1 µg/ml Hoechst 33342 DNA stain. Flow cytometry was performed with a FACSAria II (Becton, Dickinson) with a 488-nm blue laser and a 355-nm UV laser and appropriate filter sets. We collected 50,000 events per sample. Blots of DNA content versus the green channel were used to identify *B. subtilis* subpopulations, and *C. glutamicum* chromosome numbers were assessed in accordance with the standard in histograms versus the DNA amount. For data analysis, BD Accuri C6 software (BD Biosciences) or FlowJo software (Tree Star, Inc.) was used.

### Analysis of the cell cycle.

C and D periods were determined via equations relating to the amount of DNA per cell in exponential-phase cultures (68, 85), which were adapted to the *C. glutamicum* cell cycle model with double the number of chromosome equivalents at any time. Since only every second initiation is followed by a cell division, the average number of oriCs per cell (*Ī*) was defined by the equation *Ī* = 2 × 2^(*C* + *D*)/τ^, where τ is the doubling time. The term for the average number of terCs per cell (*T̅*) was adjusted accordingly with the equation *T̅* = 2 × 2^*D*/τ^. Hence, the D period was calculated as follows (the average number of oriCs [*I*] per cell [*N*] was resolved by flow cytometry) as follows *D* = {[ln(*I*/2)/*N*) × *T*_*d*_]/ln(2)} − *C*, where *T*_*d*_ is the doubling time. The equation for determination of C periods does not change upon the assumptions made above, where the oriC-to-terC (*I*/*T*) ratio was determined by marker frequency analysis as follows: *C* = [ln(*I*/*T*) × *T*_*d*_]/ln(2).

### Statistical analysis.

Analysis of variance (ANOVA) and *post hoc* tests were performed with R (86). Correlation coefficients, analysis of covariance (ANCOVA), and linear regressions were calculated with Excel and GraphPad Prism (GraphPad Software, Inc.).

## References

[1] Wang X, Montero Llopis PM, Rudner DZ 2013 Organization and segregation of bacterial chromosomes. Nat Rev Genet 14:191–203. doi:10.1038/nrg3375. 23400100 PMC3869393

[2] Quon KC, Yang B, Domian IJ, Shapiro L, Marczynski GT 1998 Negative control of bacterial DNA replication by a cell cycle regulatory protein that binds at the chromosome origin. Proc Natl Acad Sci U S A 95:120–125. doi:10.1073/pnas.95.1.120. 9419339 PMC18146

[3] McGrath PT, Iniesta AA, Ryan KR, Shapiro L, McAdams HH 2006 A dynamically localized protease complex and a polar specificity factor control a cell cycle master regulator. Cell 124:535–547. doi:10.1016/j.cell.2005.12.033. 16469700

[4] Gorbatyuk B, Marczynski GT 2005 Regulated degradation of chromosome replication proteins DnaA and CtrA in *Caulobacter crescentus*. Mol Microbiol 55:1233–1245. doi:10.1111/j.1365-2958.2004.04459.x. 15686567

[5] Lu M, Campbell JL, Boye E, Kleckner N 1994 SeqA: a negative modulator of replication initiation in *E. coli*. Cell 77:413–426. doi:10.1016/0092-8674(94)90156-2. 8011018

[6] Nievera C, Torgue JJC, Grimwade JE, Leonard AC 2006 SeqA blocking of DnaA-oriC interactions ensures staged assembly of the *E. coli* pre-RC. Mol Cell 24:581–592. doi:10.1016/j.molcel.2006.09.016. 17114060 PMC1939805

[7] Beattie TR, Reyes-Lamothe R 2015 A replisome’s journey through the bacterial chromosome. Front Microbiol 6:562. doi:10.3389/fmicb.2015.00562. 26097470 PMC4456610

[8] Katayama T, Ozaki S, Keyamura K, Fujimitsu K 2010 Regulation of the replication cycle: conserved and diverse regulatory systems for DnaA and oriC. Nat Rev Microbiol 8:163–170. doi:10.1038/nrmicro2314. 20157337

[9] Blakely G, May G, McCulloch R, Arciszewska LK, Burke M, Lovett ST, Sherratt DJ 1993 Two related recombinases are required for site-specific recombination at *dif* and *Cer* in *E. coli* K12. Cell 75:351–361. doi:10.1016/0092-8674(93)80076-Q. 8402918

[10] Aussel L, Barre FX, Aroyo M, Stasiak A, Stasiak AZ, Sherratt D 2002 FtsK is a DNA motor protein that activates chromosome dimer resolution by switching the catalytic state of the XerC and XerD recombinases. Cell 108:195–205. doi:10.1016/S0092-8674(02)00624-4. 11832210

[11] Lemon KP, Grossman AD 2000 Movement of replicating DNA through a stationary replisome. Mol Cell 6:1321–1330. doi:10.1016/S1097-2765(00)00130-1. 11163206

[12] Jensen RB, Wang SC, Shapiro L 2001 A moving DNA replication factory in *Caulobacter crescentus*. EMBO J 20:4952–4963. doi:10.1093/emboj/20.17.4952. 11532959 PMC125615

[13] Santi I, Dhar N, Bousbaine D, Wakamoto Y, McKinney JD 2013 Single-cell dynamics of the chromosome replication and cell division cycles in mycobacteria. Nat Commun 4:2470. doi:10.1038/ncomms3470. 24036848

[14] Marczynski GT 1999 Chromosome methylation and measurement of faithful, once and only once per cell cycle chromosome replication in *Caulobacter crescentus*. J Bacteriol 181:1984–1993. 10094673 10.1128/jb.181.7.1984-1993.1999 PMC93608

[15] Cooper S, Helmstetter CE 1968 Chromosome replication and the division cycle of *Escherichia coli* B/r. J Mol Biol 31:519–540. doi:10.1016/0022-2836(68)90425-7. 4866337

[16] Quinn WG, Sueoka N 1970 Symmetric replication of the *Bacillus subtilis* chromosome. Proc Natl Acad Sci U S A 67:717–723. doi:10.1073/pnas.67.2.717. 5002094 PMC283264

[17] Stokke C, Waldminghaus T, Skarstad K 2011 Replication patterns and organization of replication forks in *Vibrio cholerae*. Microbiology 157:695–708. doi:10.1099/mic.0.045112-0. 21163839

[18] Wang JD, Levin PA 2009 Metabolism, cell growth and the bacterial cell cycle. Nat Rev Microbiol 7:822–827. doi:10.1038/nrmicro2202. 19806155 PMC2887316

[19] Soppa J 2014 Polyploidy in archaea and bacteria: about desiccation resistance, giant cell size, long-term survival, enforcement by a eukaryotic host and additional aspects. J Mol Microbiol Biotechnol 24:409–419. doi:10.1159/000368855. 25732342

[20] Mendell JE, Clements KD, Choat JH, Angert ER 2008 Extreme polyploidy in a large bacterium. Proc Natl Acad Sci U S A 105:6730–6734. doi:10.1073/pnas.0707522105. 18445653 PMC2373351

[21] Sadoff HL, Shimel B, Ellis S 1979 Characterization of *Azotobacter vinelandii* deoxyribonucleic acid and folded chromosomes. J Bacteriol 138:871–877. 378943 10.1128/jb.138.3.871-877.1979 PMC218116

[22] Nagpal P, Jafri S, Reddy MA, Das HK 1989 Multiple chromosomes of *Azotobacter vinelandii*. J Bacteriol 171:3133–3138. doi:10.1128/jb.171.6.3133-3138.1989. 2785985 PMC210026

[23] Pecoraro V, Zerulla K, Lange C, Soppa J 2011 Quantification of ploidy in proteobacteria revealed the existence of monoploid, (mero-)oligoploid and polyploid species. PLoS One 6:e16392. doi:10.1371/journal.pone.0016392. 21305010 PMC3031548

[24] Hansen MT 1978 Multiplicity of genome equivalents in the radiation-resistant bacterium *Micrococcus radiodurans*. J Bacteriol 134:71–75. 649572 10.1128/jb.134.1.71-75.1978 PMC222219

[25] Griese M, Lange C, Soppa J 2011 Ploidy in cyanobacteria. FEMS Microbiol Lett 323:124–131. doi:10.1111/j.1574-6968.2011.02368.x. 22092711

[26] Hildenbrand C, Stock T, Lange C, Rother M, Soppa J 2011 Genome copy numbers and gene conversion in methanogenic archaea. J Bacteriol 193:734–743. doi:10.1128/JB.01016-10. 21097629 PMC3021236

[27] Michelsen O, Hansen FG, Albrechtsen B, Jensen PR 2010 The MG1363 and IL1403 laboratory strains of *Lactococcus lactis* and several dairy strains are diploid. J Bacteriol 192:1058–1065. doi:10.1128/JB.00900-09. 20023021 PMC2812979

[28] Wang X, Liu X, Possoz C, Sherratt DJ 2006 The two *Escherichia coli* chromosome arms locate to separate cell halves. Genes Dev 20:1727–1731. doi:10.1101/gad.388406. 16818605 PMC1522069

[29] Bates D, Kleckner N 2005 Chromosome and replisome dynamics in *E. coli*: loss of sister cohesion triggers global chromosome movement and mediates chromosome segregation. Cell 121:899–911. doi:10.1016/j.cell.2005.04.013. 15960977 PMC2973560

[30] Adachi S, Kohiyama M, Onogi T, Hiraga S 2005 Localization of replication forks in wild-type and *mukB* mutant cells of *Escherichia coli*. Mol Genet Genomics 274:264–271. doi:10.1007/s00438-005-0023-6. 16133165

[31] Viollier PH, Thanbichler M, McGrath PT, West L, Meewan M, McAdams HH, Shapiro L 2004 Rapid and sequential movement of individual chromosomal loci to specific subcellular locations during bacterial DNA replication. Proc Natl Acad Sci U S A 101:9257–9262. doi:10.1073/pnas.0402606101. 15178755 PMC438963

[32] Fogel MA, Waldor MK 2005 Distinct segregation dynamics of the two *Vibrio cholerae* chromosomes. Mol Microbiol 55:125–136. doi:10.1111/j.1365-2958.2004.04379.x. 15612922

[33] Fogel MA, Waldor MK 2006 A dynamic, mitotic-like mechanism for bacterial chromosome segregation. Genes Dev 20:3269–3282. doi:10.1101/gad.1496506. 17158745 PMC1686604

[34] Vallet-Gely I, Boccard F 2013 Chromosomal organization and segregation in *Pseudomonas aeruginosa*. PLoS Genet 9:e1003492. doi:10.1371/journal.pgen.1003492. 23658532 PMC3642087

[35] Bowman GR, Comolli LR, Zhu J, Eckart M, Koenig M, Downing KH, Moerner WE, Earnest T, Shapiro L 2008 A polymeric protein anchors the chromosomal origin/ParB complex at a bacterial cell pole. Cell 134:945–955. doi:10.1016/j.cell.2008.07.015. 18805088 PMC2745220

[36] Yamaichi Y, Bruckner R, Ringgaard S, Möll A, Cameron DE, Briegel A, Jensen GJ, Davis BM, Waldor MK 2012 A multidomain hub anchors the chromosome segregation and chemotactic machinery to the bacterial pole. Genes Dev 26:2348–2360. doi:10.1101/gad.199869.112. 23070816 PMC3475806

[37] Wang X, Montero Llopis P, Rudner DZ 2014 *Bacillus subtilis* chromosome organization oscillates between two distinct patterns. Proc Natl Acad Sci U S A 111:12877–12882. doi:10.1073/pnas.1407461111. 25071173 PMC4156703

[38] Livny J, Yamaichi Y, Waldor MK 2007 Distribution of centromere-like *parS* sites in bacteria: insights from comparative genomics. J Bacteriol 189:8693–8703. doi:10.1128/JB.01239-07. 17905987 PMC2168934

[39] Kiekebusch D, Thanbichler M 2014 Plasmid segregation by a moving ATPase gradient. Proc Natl Acad Sci U S A 111:4741–4742. doi:10.1073/pnas.1402867111. 24707041 PMC3977291

[40] Lin DC, Grossman AD 1998 Identification and characterization of a bacterial chromosome partitioning site. Cell 92:675–685. doi:10.1016/S0092-8674(00)81135-6. 9506522

[41] Murray H, Ferreira H, Errington J 2006 The bacterial chromosome segregation protein Spo0J spreads along DNA from *parS* nucleation sites. Mol Microbiol 61:1352–1361. doi:10.1111/j.1365-2958.2006.05316.x. 16925562

[42] Breier AM, Grossman AD 2007 Whole-genome analysis of the chromosome partitioning and sporulation protein Spo0J (ParB) reveals spreading and origin-distal sites on the *Bacillus subtilis* chromosome. Mol Microbiol 64:703–718. doi:10.1111/j.1365-2958.2007.05690.x. 17462018

[43] Graham TGW, Wang X, Song D, Etson CM, van Oijen AM, Rudner DZ, Loparo JJ 2014 ParB spreading requires DNA bridging. Genes Dev 28:1228–1238. doi:10.1101/gad.242206.114. 24829297 PMC4052768

[44] Leonard TA, Butler PJ, Löwe J 2005 Bacterial chromosome segregation: structure and DNA binding of the Soj dimer—a conserved biological switch. EMBO J 24:270–282. doi:10.1038/sj.emboj.7600530. 15635448 PMC545817

[45] Ptacin JL, Lee SF, Garner EC, Toro E, Eckart M, Comolli LR, Moerner WE, Shapiro L 2010 A spindle-like apparatus guides bacterial chromosome segregation. Nat Cell Biol 12:791–798. doi:10.1038/ncb2083. 20657594 PMC3205914

[46] Iniesta AA 2014 ParABS system in chromosome partitioning in the bacterium *Myxococcus xanthus*. PLoS One 9:e86897. doi:10.1371/journal.pone.0086897. 24466283 PMC3899335

[47] Lim HC, Surovtsev IV, Beltran BG, Huang F, Bewersdorf J, Jacobs-Wagner C 2014 Evidence for a DNA-relay mechanism in ParABS-mediated chromosome segregation. eLife 3:e02758. doi:10.7554/eLife.02758. 24859756 PMC4067530

[48] Hwang LC, Vecchiarelli AG, Han YW, Mizuuchi M, Harada Y, Funnell BE, Mizuuchi K 2013 ParA-mediated plasmid partition driven by protein pattern self-organization. EMBO J 32:1238–1249. doi:10.1038/emboj.2013.34. 23443047 PMC3642677

[49] Vecchiarelli AG, Hwang LC, Mizuuchi K 2013 Cell-free study of F plasmid partition provides evidence for cargo transport by a diffusion-ratchet mechanism. Proc Natl Acad Sci U S A 110:E1390–E1397. doi:10.1073/pnas.1302745110. PMC3625265 23479605

[50] Lewis RA, Bignell CR, Zeng W, Jones AC, Thomas CM 2002 Chromosome loss from par mutants of *Pseudomonas putida* depends on growth medium and phase of growth. Microbiology 148:537–548. doi:10.1099/00221287-148-2-537. 11832517

[51] Donovan C, Schwaiger A, Krämer R, Bramkamp M 2010 Subcellular localization and characterization of the ParAB system from *Corynebacterium glutamicum*. J Bacteriol 192:3441–3451. doi:10.1128/JB.00214-10. 20435732 PMC2897671

[52] Jakimowicz D, Brzostek A, Rumijowska-Galewicz A, Żydek P, Dołzbłasz A, Smulczyk-Krawczyszyn A, Zimniak T, Wojtasz L, Zawilak-Pawlik A, Kois A, Dziadek J, Zakrzewska-Czerwińska J 2007 Characterization of the mycobacterial chromosome segregation protein ParB and identification of its target in *Mycobacterium smegmatis*. Microbiology 153:4050–4060. doi:10.1099/mic.0.2007/011619-0. 18048919

[53] Ireton K, Gunther NW, Grossman AD 1994 Spo0J is required for normal chromosome segregation as well as the initiation of sporulation in *Bacillus subtilis*. J Bacteriol 176:5320–5329. doi:10.1128/jb.176.17.5320-5329.1994. 8071208 PMC196717

[54] Ginda K, Bezulska M, Ziółkiewicz M, Dziadek J, Zakrzewska-Czerwińska J, Jakimowicz D 2013 ParA of *Mycobacterium smegmatis* co-ordinates chromosome segregation with the cell cycle and interacts with the polar growth determinant DivIVA. Mol Microbiol 87:998–1012. doi:10.1111/mmi.12146. 23289458

[55] Mohl DA, Easter J Jr., Gober JW 2001 The chromosome partitioning protein, ParB, is required for cytokinesis in *Caulobacter crescentus*. Mol Microbiol 42:741–755. doi:10.1046/j.1365-2958.2001.02643.x. 11722739

[56] Charaka VK, Misra HS 2012 Functional characterization of the role of the chromosome I partitioning system in genome segregation in *Deinococcus radiodurans*. J Bacteriol 194:5739–5748. doi:10.1128/JB.00610-12. 22843847 PMC3486093

[57] WHO 2016 Global tuberculosis report 2016. World Health Organization, Geneva, Switzerland http://apps.who.int/iris/bitstream/10665/250441/1/9789241565394-eng.pdf?ua=1

[58] Wendisch VF, Jorge JMP, Pérez-García F, Sgobba E 2016 Updates on industrial production of amino acids using *Corynebacterium glutamicum*. World J Microbiol Biotechnol 32:105. doi:10.1007/s11274-016-2060-1. 27116971

[59] Donovan C, Schauss A, Krämer R, Bramkamp M 2013 Chromosome segregation impacts on cell growth and division site selection in *Corynebacterium glutamicum*. PLoS One 8:e55078. doi:10.1371/journal.pone.0055078. 23405112 PMC3566199

[60] Donovan C, Sieger B, Krämer R, Bramkamp M 2012 A synthetic *Escherichia coli* system identifies a conserved origin tethering factor in actinobacteria. Mol Microbiol 84:105–116. doi:10.1111/j.1365-2958.2012.08011.x. 22340668

[61] Donovan C, Bramkamp M 2014 Cell division in *Corynebacterineae*. Front Microbiol 5:132. doi:10.3389/fmicb.2014.00132. 24782835 PMC3989709

[62] Harms A, Treuner-Lange A, Schumacher D, Søgaard-Andersen L 2013 Tracking of chromosome and replisome dynamics in *Myxococcus xanthus* reveals a novel chromosome arrangement. PLoS Genet 9:e1003802. doi:10.1371/journal.pgen.1003802. 24068967 PMC3778016

[63] Thanbichler M, Shapiro L 2006 MipZ, a spatial regulator coordinating chromosome segregation with cell division in *Caulobacter*. Cell 126:147–162. doi:10.1016/j.cell.2006.05.038. 16839883

[64] Santi I, McKinney JD 2015 Chromosome organization and replisome dynamics in *Mycobacterium smegmatis*. mBio 6:e01999-14. doi:10.1128/mBio.01999-14. 25691587 PMC4337562

[65] Trojanowski D, Ginda K, Pióro M, Hołówka J, Skut P, Jakimowicz D, Zakrzewska-Czerwińska J 2015 Choreography of the *Mycobacterium* replication machinery during the cell cycle. mBio 6:e02125-14. doi:10.1128/mBio.02125-14. 25691599 PMC4337567

[66] Sieger B, Schubert K, Donovan C, Bramkamp M 2013 The lipid II flippase RodA determines morphology and growth in *Corynebacterium glutamicum*. Mol Microbiol 90:966–982. doi:10.1111/mmi.12411. 24118443

[67] Fossum S, Crooke E, Skarstad K 2007 Organization of sister origins and replisomes during multifork DNA replication in *Escherichia coli*. EMBO J 26:4514–4522. doi:10.1038/sj.emboj.7601871. 17914458 PMC2063475

[68] Hill NS, Kadoya R, Chattoraj DK, Levin PA 2012 Cell size and the initiation of DNA replication in bacteria. PLoS Genet 8:e1002549. doi:10.1371/journal.pgen.1002549. 22396664 PMC3291569

[69] Badrinarayanan A, Le TB, Laub MT 2015 Bacterial chromosome organization and segregation. Annu Rev Cell Dev Biol 31:171–199. doi:10.1146/annurev-cellbio-100814-125211. 26566111 PMC4706359

[70] Randich AM, Brun YV 2015 Molecular mechanisms for the evolution of bacterial morphologies and growth modes. Front Microbiol 6:580. doi:10.3389/fmicb.2015.00580. 26106381 PMC4460556

[71] Jensen RB, Shapiro L 1999 The *Caulobacter crescentus smc* gene is required for cell cycle progression and chromosome segregation. Proc Natl Acad Sci U S A 96:10661–10666. doi:10.1073/pnas.96.19.10661. 10485882 PMC17939

[72] Lemon KP, Grossman AD 1998 Localization of bacterial DNA polymerase: evidence for a factory model of replication. Science 282:1516–1519. doi:10.1126/science.282.5393.1516. 9822387

[73] Neumeyer A, Hübschmann T, Müller S, Frunzke J 2013 Monitoring of population dynamics of *Corynebacterium glutamicum* by multiparameter flow cytometry. Microb Biotechnol 6:157–167. doi:10.1111/1751-7915.12018. 23279937 PMC3917458

[74] Potts M 1994 Desiccation tolerance of prokaryotes. Microbiol Rev 58:755–805. 7854254 10.1128/mr.58.4.755-805.1994 PMC372989

[75] Ochrombel I, Ott L, Krämer R, Burkovski A, Marin K 2011 Impact of improved potassium accumulation on pH homeostasis, membrane potential adjustment and survival of *Corynebacterium glutamicum*. Biochim Biophys Acta 1807:444–450. doi:10.1016/j.bbabio.2011.01.008. 21295539

[76] Miyamoto-Shinohara Y, Sukenobe J, Imaizumi T, Nakahara T 2008 Survival of freeze-dried bacteria. J Gen Appl Microbiol 54:9–24. doi:10.2323/jgam.54.9. 18323678

[77] Gilichinsky DA, Vorobyova EA, Erokhina LG, Fyordorov-Davydov DG, Chaikovskaya NR, Fyordorov-Dayvdov DG 1992 Long-term preservation of microbial ecosystems in permafrost. Adv Space Res 12:255–263. doi:10.1016/0273-1177(92)90180-6. 11538146

[78] Schäfer A, Tauch A, Jäger W, Kalinowski J, Thierbach G, Pühler A 1994 Small mobilizable multi-purpose cloning vectors derived from the *Escherichia coli* plasmids pK18 and pK19: selection of defined deletions in the chromosome of *Corynebacterium glutamicum*. Gene 145:69–73. doi:10.1016/0378-1119(94)90324-7. 8045426

[79] Nottebrock D, Meyer U, Krämer R, Morbach S 2003 Molecular and biochemical characterization of mechanosensitive channels in *Corynebacterium glutamicum*. FEMS Microbiol Lett 218:305–309. doi:10.1111/j.1574-6968.2003.tb11533.x. 12586408

[80] Keilhauer C, Eggeling L, Sahm H 1993 Isoleucine synthesis in *Corynebacterium glutamicum*: molecular analysis of the *ilvB*-*ilvN*-*ilvC* operon. J Bacteriol 175:5595–5603. doi:10.1128/jb.175.17.5595-5603.1993. 8366043 PMC206616

[81] Schindelin J, Arganda-Carreras I, Frise E, Kaynig V, Longair M, Pietzsch T, Preibisch S, Rueden C, Saalfeld S, Schmid B, Tinevez JY, White DJ, Hartenstein V, Eliceiri K, Tomancak P, Cardona A 2012 Fiji: an open-source platform for biological-image analysis. Nat Methods 9:676–682. doi:10.1038/nmeth.2019. 22743772 PMC3855844

[82] Rasmussen R 2001 Quantification on the LightCycler, p 21–34. *In* Meuer S, Wittwer C, Nakagawara K (ed), Rapid cycle real-time PCR methods and applications. Springer, Berlin, Germany.

[83] Livak KJ, Schmittgen TD 2001 Analysis of relative gene expression data using real-time quantitative PCR and the 2(-Delta Delta C_(T)_) method. Methods 25:402–408. doi:10.1006/meth.2001.1262. 11846609

[84] Hammes F, Berney M, Wang Y, Vital M, Köster O, Egli T 2008 Flow-cytometric total bacterial cell counts as a descriptive microbiological parameter for drinking water treatment processes. Water Res 42:269–277. doi:10.1016/j.watres.2007.07.009. 17659762

[85] Bremer H, Churchward G 1977 An examination of the Cooper-Helmstetter theory of DNA replication in bacteria and its underlying assumptions. J Theor Biol 69:645–654. doi:10.1016/0022-5193(77)90373-3. 607026

[86] The R Development Core Team 2014 R: a language and environment for statistical computing. R Foundation for Statistical Computing, Vienna, Austria http://www.R-project.org/.

[87] Nair N, Dziedzic R, Greendyke R, Muniruzzaman S, Rajagopalan M, Madiraju MV 2009 Synchronous replication initiation in novel *Mycobacterium tuberculosis dnaA* cold-sensitive mutants. Mol Microbiol 71:291–304. doi:10.1111/j.1365-2958.2008.06523.x. 19019143 PMC2733369

[88] Zusman D, Rosenberg E 1970 DNA cycle of *Myxococcus xanthus*. J Mol Biol 49:609–619. doi:10.1016/0022-2836(70)90285-8. 5453346

[89] Dingwall A, Shapiro L 1989 Rate, origin, and bidirectionality of *Caulobacter* chromosome replication as determined by pulsed-field gel electrophoresis. Proc Natl Acad Sci U S A 86:119–123. doi:10.1073/pnas.86.1.119. 2911562 PMC286415

[90] Rasmussen T, Jensen RB, Skovgaard O 2007 The two chromosomes of *Vibrio cholerae* are initiated at different time points in the cell cycle. EMBO J 26:3124–3131. doi:10.1038/sj.emboj.7601747. 17557077 PMC1914095

[91] Sharpe ME, Hauser PM, Sharpe RG, Errington J 1998 *Bacillus subtilis* cell cycle as studied by fluorescence microscopy: constancy of cell length at initiation of DNA replication and evidence for active nucleoid partitioning. J Bacteriol 180:547–555. 9457856 10.1128/jb.180.3.547-555.1998 PMC106920

[92] Kubitschek HE, Freedman ML 1971 Chromosome replication and the division cycle of *Escherichia coli* B-r. J Bacteriol 107:95–99. 4935333 10.1128/jb.107.1.95-99.1971 PMC246890

[93] Michelsen O, Teixeira de Mattos MJ, Jensen PR, Hansen FG 2003 Precise determinations of C and D periods by flow cytometry in *Escherichia coli* K-12 and B/r. Microbiology 149:1001–1010. doi:10.1099/mic.0.26058-0. 12686642

[94] Tanner NA, Loparo JJ, Hamdan SM, Jergic S, Dixon NE, van Oijen AM 2009 Real-time single-molecule observation of rolling-circle DNA replication. Nucleic Acids Res 37:e27. doi:10.1093/nar/gkp006. 19155275 PMC2651787

