# Supplementary figures and images for: Novel Chromosome Organization Pattern in Actinomycetales—Overlapping Replication Cycles Combined with Diploidy
Source: mBio. 2017 Jun 6;8(3):e00511-17. doi: 10.1128/mBio.00511-17 (PMC5461407; doi:10.1128/mBio.00511-17)

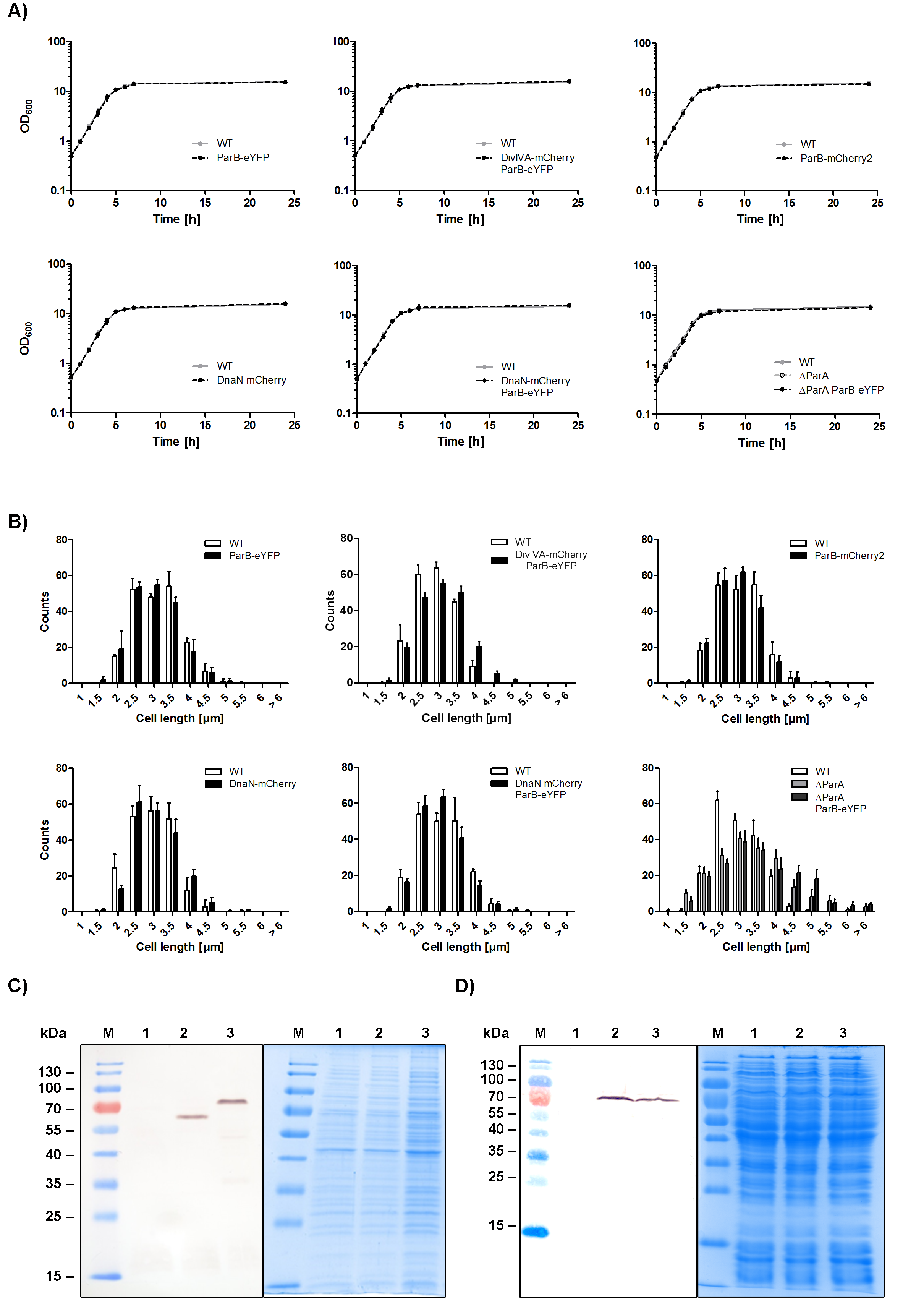

Supplement: FIG S1 [file mbo003173336sf1.tif]

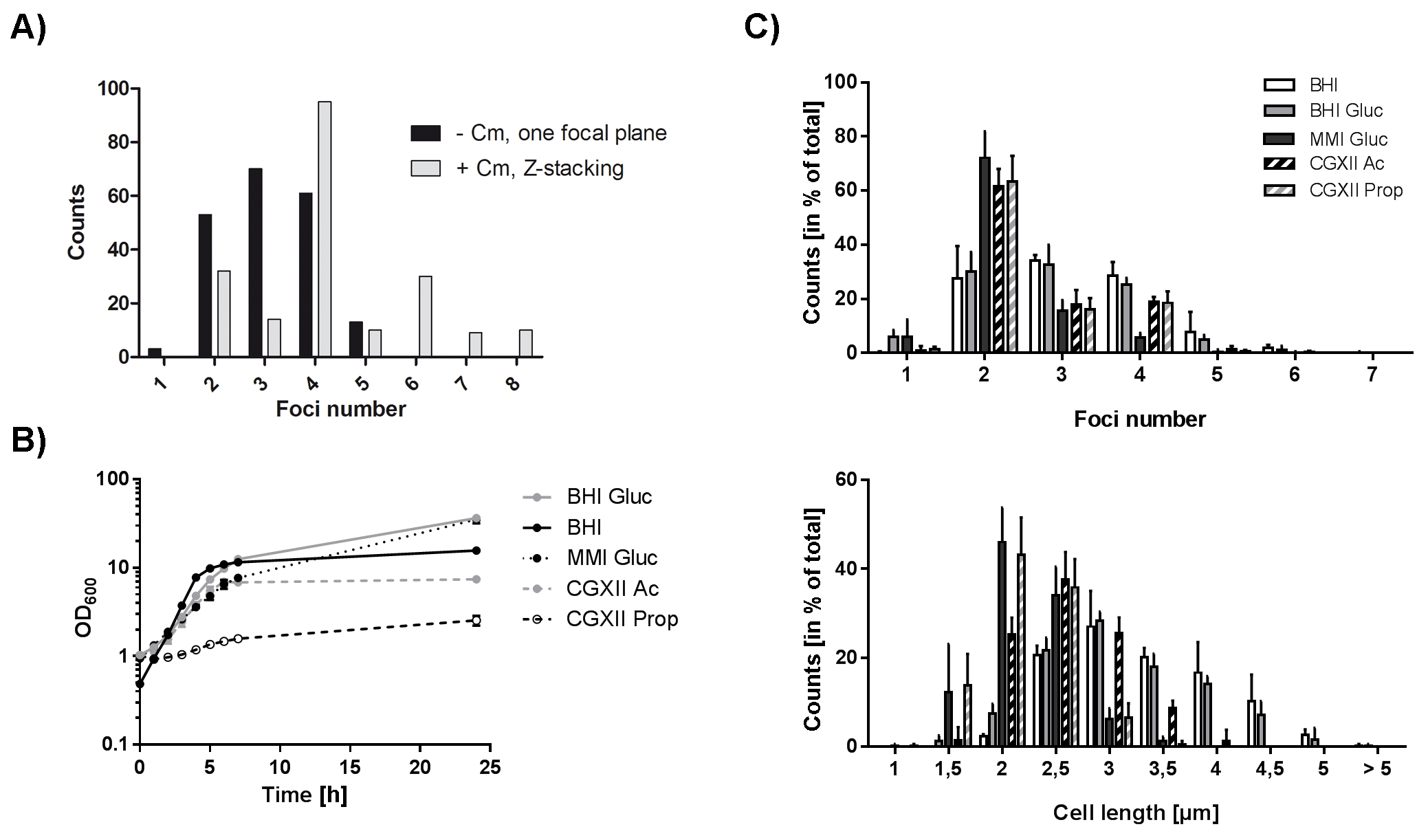

Supplement: FIG S2 [file mbo003173336sf2.tif]

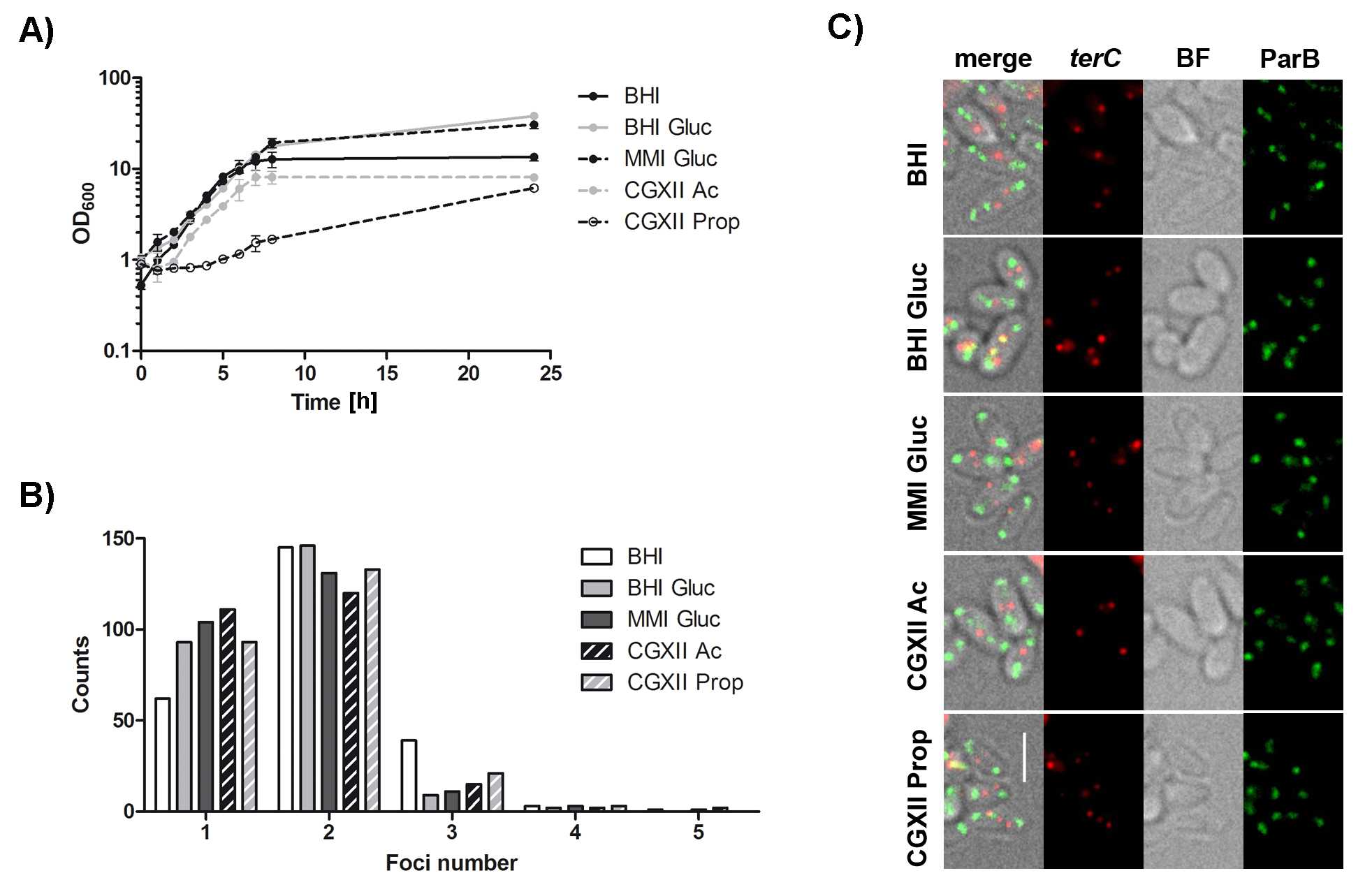

Supplement: FIG S3 [file mbo003173336sf3.tif]

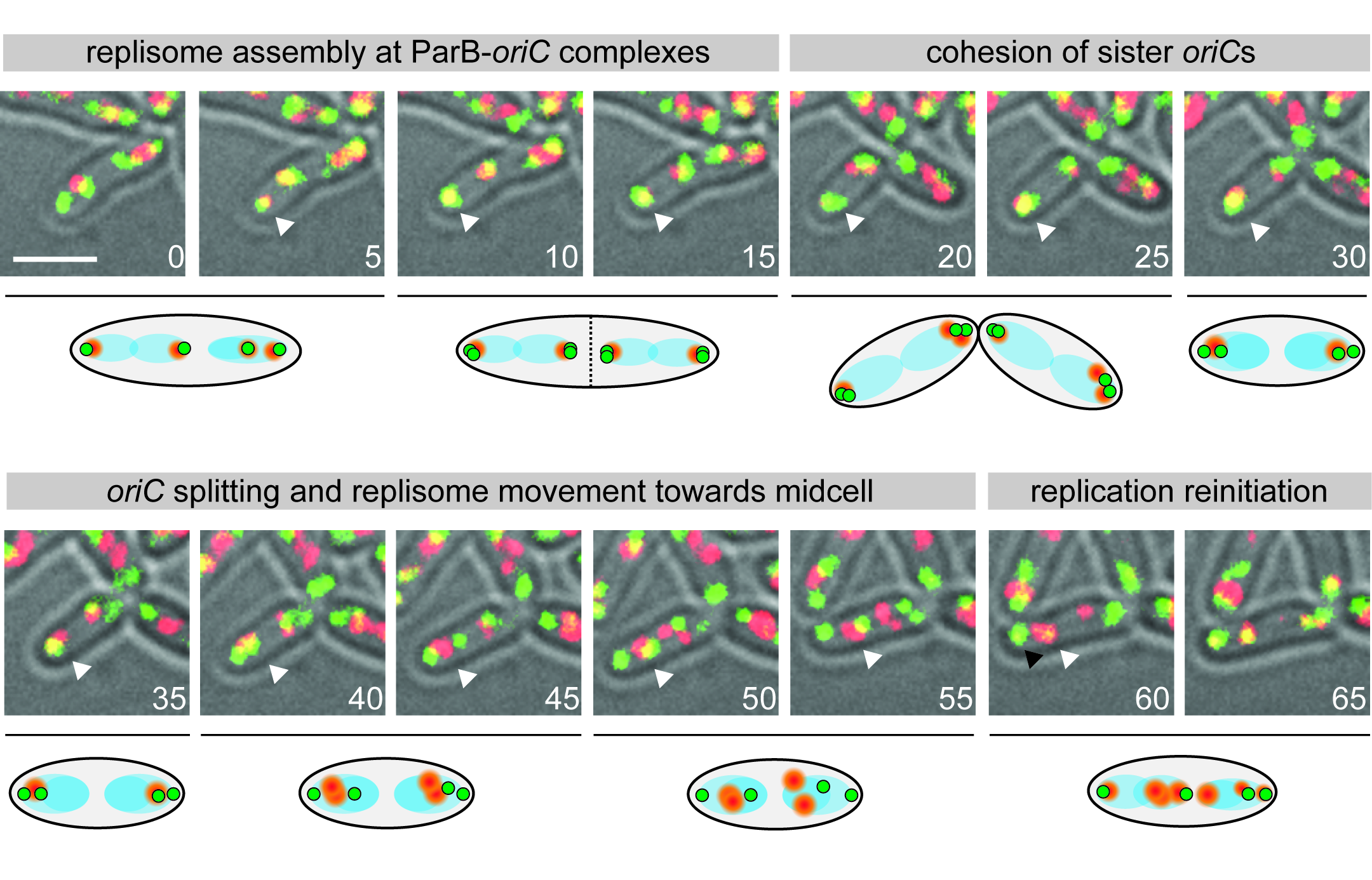

Supplement: FIG S4 [file mbo003173336sf4.tif]

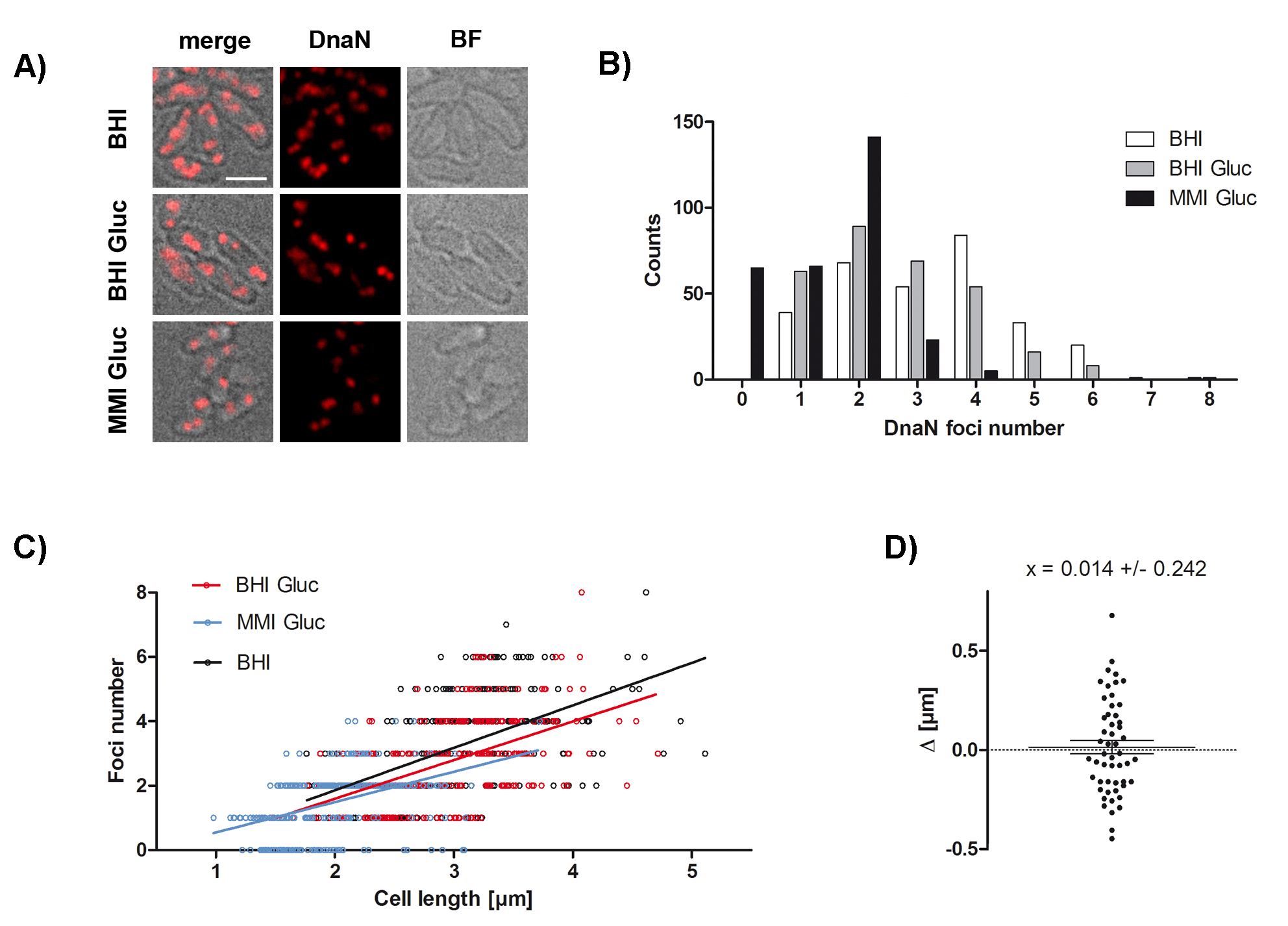

Supplement: FIG S5 [file mbo003173336sf5.tif]

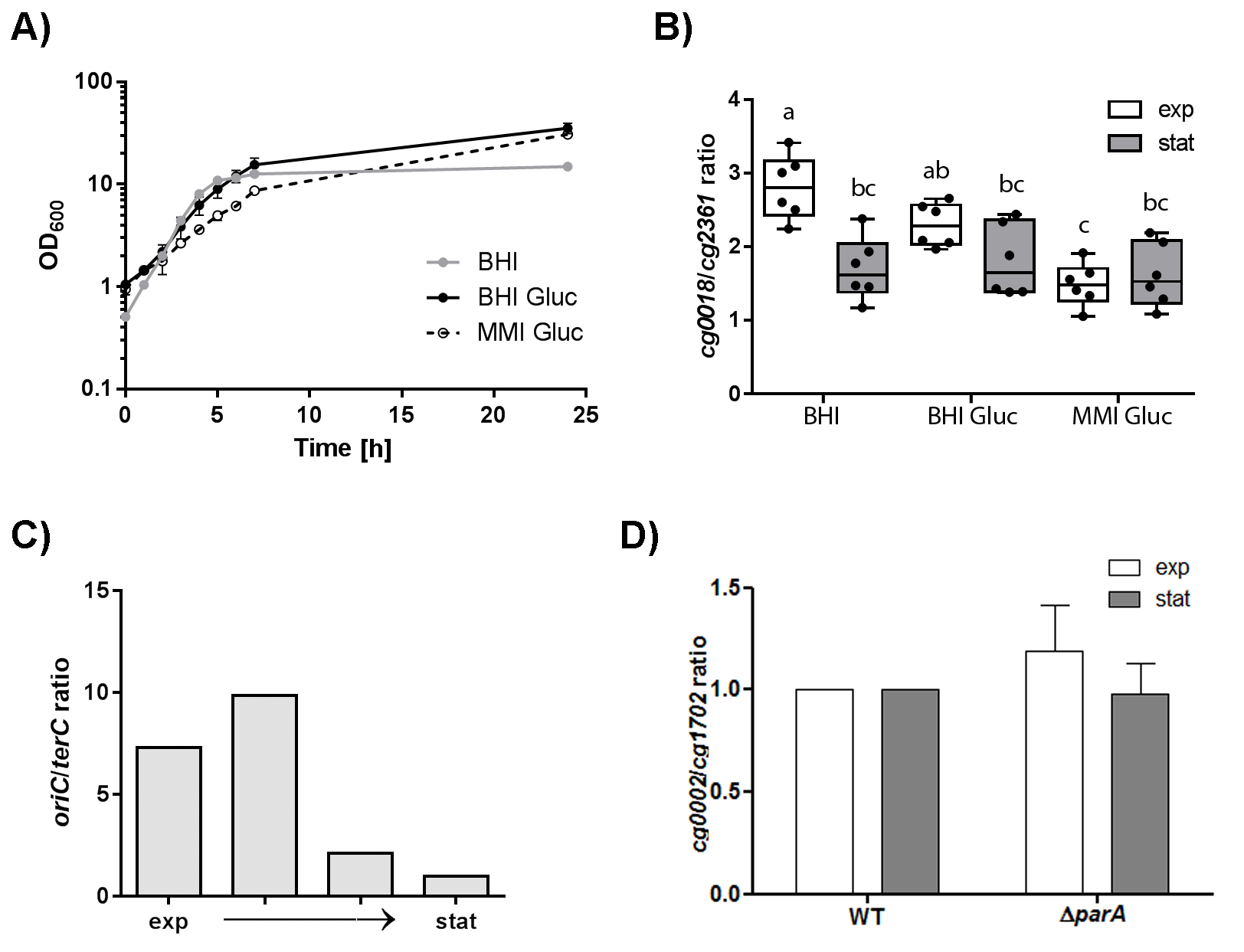

Supplement: FIG S6 [file mbo003173336sf6.tif]

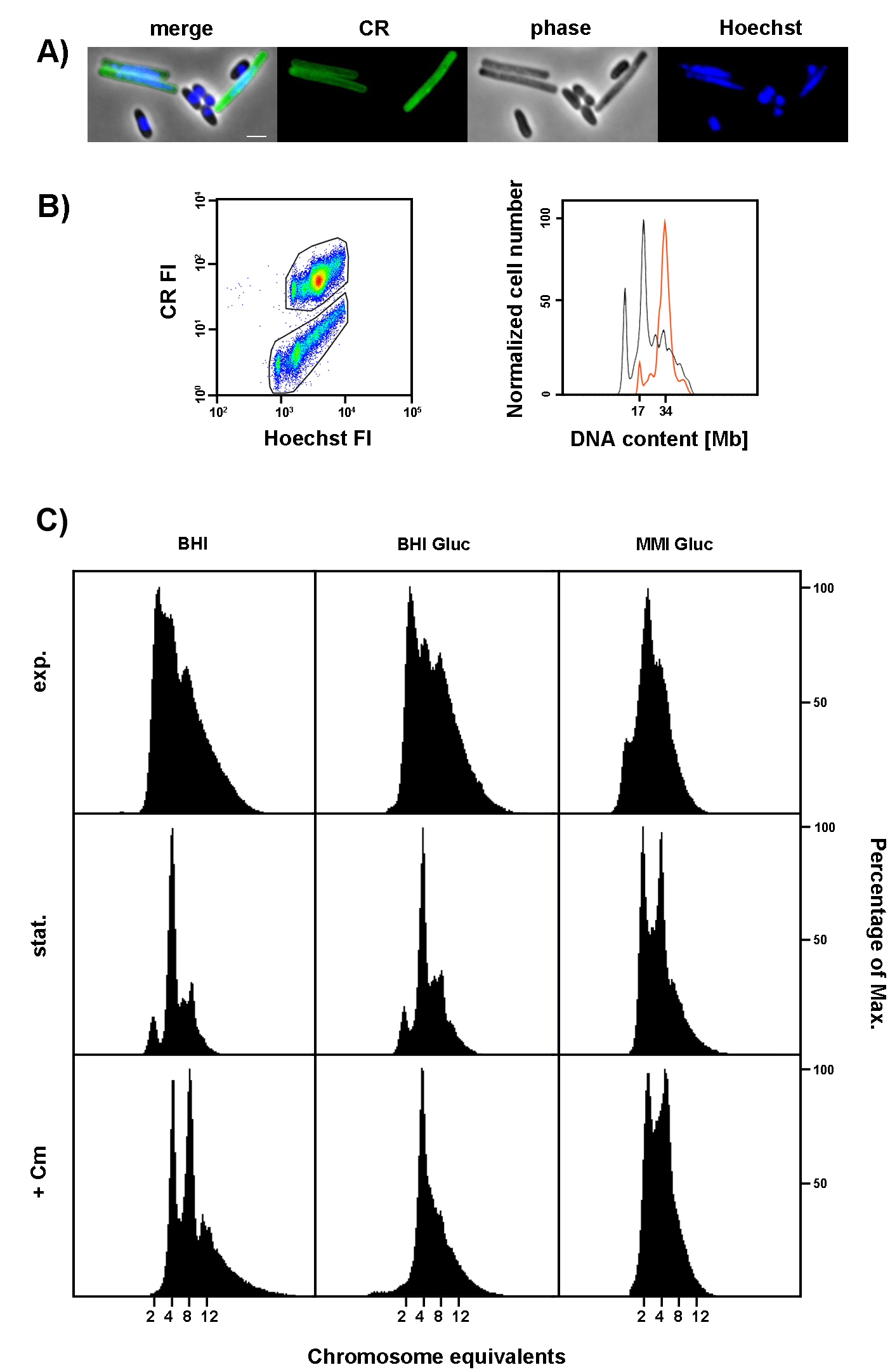

Supplement: FIG S7 [file mbo003173336sf7.tif]
