# Supplementary material for: Novel Chromosome Organization Pattern in Actinomycetales—Overlapping Replication Cycles Combined with Diploidy
Source: mBio. 2017 Jun 6;8(3):e00511-17. doi: 10.1128/mBio.00511-17 (PMC5461407; doi:10.1128/mBio.00511-17)
Supplement: TEXT S1 [file mbo003173336st1.docx]

**Text S1.**

Table S1. Oligonucleotides used in this study.

| Oligonucleotides  Strain constructions | Sequence 5'–3' | | Restriction sites ^a^ |
| --- | --- | --- | --- |
| ParB-Hind-up-F | CATAAGCTTAGCTGAATCCTTTGGAAG | | HindIII |
| ParB-Sal-up-R | CATGTCGACTTGGCCCTGGATCAAGGA | | SalI |
| ParB-XbaI-D-F | CATTCTAGATAATTTTAAGTTTGGCGC | | XbaI |
| ParB-Bam-D-R | CATGGATCCCCTCCACATCAATCAGGC | | BamHI |
| eYFP-SalI-F | CATGTCGACATGGTGAGCAAGGGCGAG | | SalI |
| eYFP-XbaI-R | CATTCTAGACTTGTACAGCTCGTCCAT | | XbaI |
| mCherry2-SalI-F | ATAGTCGACATGGTCAGCAAGG | | SalI |
| mCherry2-XbaI-R | ATATCTAGAGGATCCTGAGCCG | | XbaI |
| ParB-N-ter-SalI-F | CAGGTCGACATGGCTCAGAACAAGGGTTCC | | SalI |
| ParB-PstI-800D-R | CAGCTGCAGCCAACCCCGATGACCTGG | | PstI |
| DnaN-Hind-up-F | CATAAGCTTGGTTGGCCGCGAAGGACT | | HindIII |
| DnaN-SphI-up-R | CATGCATGCGCCTGGCAGGCGCACTGG | | SphI |
| DnaN-XbaI-D-F | CATTCTAGATAAACACAAAAGTTTCAC | | XbaI |
| DnaN-BamHI-D-R | CATGGATCCTCTGCTGGCTCGCCTTTG | | BamHI |
| mCherry-SalI-F | CATGTCGACATGGTGAGCAAGGGCGAG | | SalI |
| mCherry-XbaI-R | GCGTCTAGATTACTTGTACAGCTCGTC | | XbaI |
| DnaN-N-ter-F | ATGGAGTCACAAAACGTGTCCTTC | |  |
| DnaN-Bam-700D-R | CATGGATCCGGCGTGCCAACTGG | | BamHI |
| Int-HindIII-up-F | ATAAAGCTTATTACCAGGAGCGCC | | HindIII |
| Int-PstI-up-R | ATACTGCAGCCGTTTGTTATGTG | | PstI |
| Int-EcoRI-D-F | CGCGAATTCAACGAAACAGTCTTGACC | | EcoRI |
| Int-NheI-D-R | ATAGCTAGCGGCGGCATCGTCAC | | NheI |
| Int-700up-F | AGCAGATAAAGTTCCAATTGAATGG | |  |
| Int-700D-R | TTTTCCCAGAACCAGCACC | |  |
| LacI-SalI-F | ATAGTCGACAGGAGGAATTCACCAT | | SalI |
| CFP-KpnI-R | ATAGGTACCTTACTTGTACAGCTCGTCC | | KpnI |
| Oligonucleotides  Marker frequency analysis | **Sequence 5'–3'** | **Organism and chromosomal binding region** | |
| Cg0002-165-F | TTTTGGGGAGTTGTGCACAG | *C. glutamicum*, cg0002 | |
| Cg0002-165-R | GGGTTGTGCAGGGATTTTGT | *C. glutamicum*, cg0002 | |
| Cg1702-159-F | TGAAGCTATCCTCAACGGCA | *C. glutamicum*, cg1702 | |
| Cg1702-159-R | TAACCAATCGCGATGCCTTG | *C. glutamicum*, cg1702 | |
| DivIVA-153-F | CTACAACGAAGACGAGGT | *C. glutamicum*, cg1702 | |
| DivIVA-153-R | GCAGTTGAGGAACTAGCA | *C. glutamicum*, cg0018 | |
| Cg0018-262-F | CCTGCTCAGAATGAAACC | c *C. glutamicum*, g0018 | |
| Cg0018-262-R | AGCTGCTACTACTTGGGC | *C. glutamicum*, cg0018 | |
| Bsu-oriC-F | GATCAATCGGGGAAAGTGTG | *B. subtilis*, 5’ site: BSU00010, 3’ site: BSU00020 | |
| Bsu-oriC-R | GTAGGGCCTGTGGATTTGTG | *B. subtilis*, 5’ site: BSU00010, 3’ site: BSU00020 | |
| Bsu-terC-F | TCCATATCCTCGCTCCTACG | *B. subtilis*, 5’ site: BSU19200, 3’ site: BSU19210 | |
| Bsu-terC-R | ATTCTGCTGATGTGCAATGG | *B. subtilis*, 5’ site: BSU19200 3’ site: BSU19210 | |

^a^ Restriction sites are underlined.

Table S2. Bacterial strains and plasmids used in this study.

| Strain | | Characteristics | References/  Source |
| --- | --- | --- | --- |
| *E. coli DH5α* | | F^-^ ф80*lacZ*ΔM15 (*lacZYA-argF*)*U169 recA1 endA1 hsdR17*(r_K_^-^ m_K_^+^) *supE44* *phoA thi-1 gyrA96 relA1* λ^-^ | Invitrogen |
| *B. subtilis 168* | | *trpC2* | Laboratory collection |
| *C. glutamicum* RES 167 | | Restriction-deficient mutant, otherwise considered wild type | ([1](#_ENREF_1)) |
| *C. glutamicum parB::parB-eYFP* | | RES167 derivative, *parB*-*eYFP* | This study |
| *C. glutamicum parB::parB-mCherry2* | | RES167 derivative, *parB*-*mCherry2* | This study |
| *C. glutamicum ΔparA* | | RES167 derivative, *ΔparA* | ([2](#_ENREF_2)) |
| *C. glutamicum ΔparA parB::parB-eYFP* | | RES167 derivative, *ΔparA, parB*-*eYFP* | This study |
| *C. glutamicum divIVA::divIVA-mCherry* | | RES167 derivative, *divIVA*-*mCherry* | ([3](#_ENREF_3)) |
| *C. glutamicum divIVA::divIVA-mCherry parB::parB-eYFP* | | RES167 derivative, *divIVA*-*mCherry*, *parB*-*eYFP* | This study |
| *C. glutamicum dnaN::dnaN-mCherry* | | RES167 derivative, *dnaN*-*mCherry* | This study |
| *C. glutamicum parB::parB-eYFP dnaN::dnaN-mCherry* | | RES167 derivative, *dnaN*-*mCherry*, *parB*-*eYFP* | This study |
| *C. glutamicum parB::parB-eYFP*, *int∷lacO* | | RES167 derivative, *parB*-*eYFP*, *int∷lacO* | This study |
| *C. glutamicum parB::parB-eYFP*, *int∷lacO*, pCLTON1PamtR-*lacI-CFP* | | RES167 derivative, *parB*-*eYFP*, *int∷lacO*, tet-inducible LacI-CFP expression | This study |
| Plasmids | **Characteristics** | | **Reference/**  **Source** |
| pK19mobsacB | Integration vector, *ori* pUC, Km^r^, *mob sac* | | ([4](#_ENREF_4)) |
| pK19mobsacB-parB-eYFP | Integration vector, *ori* pUC, Km^r^, *mob sac, parB*-*eYFP* | | This study |
| pK19mobsacB-parB-mCherry2 | Integration vector, *ori* pUC, Km^r^, *mob sac, parB*-*mCherry2* | | This study |
| pK19mobsacB-DnaN-mCherry | Integration vector, *ori* pUC, Km^r^, *mob sac, dnaN-mCherry* | | This study |
| pLAU43 | Km^R^, Amp^R^, pMB1 *ori*, *lacO* | | ([5](#_ENREF_5)) |
| pLAU53 | araBp-lacI-eCFP, araBp-tetR-eYFP, pMB1 *ori*, | | ([5](#_ENREF_5)) |
| pK19mobsacB-int∷lacO | Integration vector, *ori* pUC, Km^r^, *mob sac, int∷lacO* | | This study |
| pEKEx2 | *E. coli*-*C. glutamicum* shuttle expression vector, P_tac_, lacI_q_, Km^R^, pBL1 *oriV_C.g._*_,_ pUC18 *oriV_E.c._* | | ([6](#_ENREF_6)) |
| pCLTON1PamtR | Modified pCLTON1 expression vector | | Gerd Seibold |
| pCLTON1PamtR-*lacI-CFP* | pCLTON1PamtR, *lacI-CFP* | | This study |

**References**

1. Tauch, A., Kirchner, O., Löffler, B., Götker, S., Pühler, A. and Kalinowski, J. (2002) Efficient electrotransformation of corynebacterium diphtheriae with a mini-replicon derived from the Corynebacterium glutamicum plasmid pGA1. *Current microbiology*, **45**, 362-367.

2. Donovan, C., Schwaiger, A., Krämer, R. and Bramkamp, M. (2010) Subcellular localization and characterization of the ParAB system from *Corynebacterium glutamicum*. *Journal of bacteriology*, **192**, 3441-3451.

3. Donovan, C., Sieger, B., Krämer, R. and Bramkamp, M. (2012) A synthetic *Escherichia coli* system identifies a conserved origin tethering factor in Actinobacteria. *Molecular microbiology*, **84**, 105-116.

4. Schäfer, A., Tauch, A., Jäger, W., Kalinowski, J., Thierbach, G. and Pühler, A. (1994) Small mobilizable multi-purpose cloning vectors derived from the *Escherichia coli* plasmids pK18 and pK19: selection of defined deletions in the chromosome of *Corynebacterium glutamicum*. *Gene*, **145**, 69-73.

5. Lau, I.F., Filipe, S.R., Soballe, B., Økstad, O.A., Barre, F.X. and Sherratt, D.J. (2003) Spatial and temporal organization of replicating *Escherichia coli* chromosomes. *Molecular microbiology*, **49**, 731-743.

6. Eikmanns, B.J., Kleinertz, E., Liebl, W. and Sahm, H. (1991) A family of *Corynebacterium glutamicum*/*Escherichia coli* shuttle vectors for cloning, controlled gene expression, and promoter probing. *Gene*, **102**, 93-98.
